# Sound, stress, and health in youth orchestras: feasibility of a multimodal psychophysiological health promotion program

**DOI:** 10.3389/fpsyg.2026.1742780

**Published:** 2026-02-20

**Authors:** Matthias Bertsch, Marik Roos, Tristan Leitz, Mona Smale, Christoph Reuter

**Affiliations:** 1 Centre of Music Physiology, University of Music and Performing Arts Vienna, Wien, Austria; 2 SInES (Space for Interdisciplinary Experiments on Sound), University of Vienna, Wien, Austria

**Keywords:** health promotion, hearing protection, music performance anxiety (MPA), orchestral musicians, playing-related musculoskeletal disorders (PRMD), virtual reality exposure training (VRET), wearable stress monitoring, youth orchestras

## Abstract

**Background:**

Young orchestral musicians face significant health challenges, including elevated psychological stress, playing-related musculoskeletal pain, and exposure to high sound pressure levels (frequently 90–100 dB_SPL_) that pose risks to hearing health. Traditional music education often lacks systematic health promotion and preventative strategies, resulting in a high prevalence of health issues that can compromise long-term performance and well-being. This study addresses the need for a comprehensive, evidence-based program to promote sustainable health and resilience practices in young orchestral musicians.

**Research question:**

Can a multimodal, integrated health promotion program combining acoustic, physiological, psychological, and behavioral interventions be feasibly and acceptably implemented into the intense rehearsal culture of European youth orchestras and how do participating musicians engage with the program’s components?

**Methods:**

A mixed-methods design was implemented during multi-site summer and autumn camps involving 136 musicians (aged 14–27) from two European youth orchestras. The program consisted of six interlinked modules: (1) orchestral soundscape mapping via 16 microphones to document sound pressure levels, (2) practical interventions including daily warm-ups, ‘BodyFit,’ and mental training workshops, (3) audiometric screening of 77 participants, (4) Virtual Reality Exposure Training (VRET) for performance anxiety, (5) continuous psychophysiological stress monitoring using EmbracePlus smartwatches on 15 musicians, and (6) quantitative pre- and post-camp surveys.

**Results:**

Acoustic mapping confirmed significant spatial variation in sound exposure, with peak levels of 88–100 dB_SPL_, particularly among brass and percussion players. Audiometric screening revealed that while most thresholds were normal, an early high-frequency mild hearing loss was already evident among brass players, highlighting potential hearing-related vulnerabilities. Wearable monitoring successfully identified individual stress patterns that were temporally associated with challenging musical passages and emotional climaxes. Post-intervention surveys indicated short-term, self-reported improvements in stress management and ergonomic awareness among participants who took part the interventions. Overall, the program showed a high degree of uptake, with over 90% engagement across modules, although inconsistent use of hearing protection remained an issue (40% reported inconsistent use).

**Conclusion:**

The study demonstrates the feasibility and high degree of uptake of a multimodal health promotion framework within the context of a youth orchestra. By combining objective measurements with practical, evidence-based training, the program demonstrates how health-oriented practices can be integrated into orchestral rehearsal culture, and it provides a concrete starting point for future controlled intervention studies.

## 1 Introduction

Young orchestral musicians are exposed to a wide range of challenges that extend beyond achieving technical mastery. High-level ensemble performance frequently requires considerable physical demands, acoustically environments, and elevated psychological stress, all of which may compromise health as well as artistic performance (Ackermann et al., 2014; Détári et al., 2020; Gembris et al., 2018). Two decades ago, Britsch (2005) highlighted that performance-related pain and anxiety were widespread among youth orchestra musicians; furthermore, systematic prevention and health education were largely absent. Prolonged exposure to intense sound pressure levels, combined with the psychological pressures of public performance and career preparation, raises concerns about the long-term well-being of young musicians (Gembris et al., 2018). Traditional training environments often insufficiently address the physiological and psychological resilience required for sustainable performance, despite growing evidence of its importance for coping with perfectionism and mental strain among musicians (Arbinaga, 2023; Kegelaers et al., 2020). Recent large-scale data from the “Jugend musiziert” competition in Germany indicate that up to 76% of high-performing young musicians report playing-related pain (Gembris et al., 2020). Playing musical instruments places highly specific physical and psychological demands on their bodies and minds. When the balance between load and recovery is lost, musicians face an elevated risk of developing playing-related musculoskeletal disorders (PRMD) and other related health problems (Spahn, 2022).

Early epidemiological research has already documented high rates of playing-related musculoskeletal complaints among both student and professional musicians (Fry, 1986; Fishbein et al., 1988; Zaza, 1998; Larsson et al., 1993). In a German survey of 1,862 professionals and pre-professionals, nearly half of respondents under the age of 30 reported playing-related pain in the preceding three months, and over a third indicated chronic musculoskeletal problems (Gembris et al., 2018). Mental health issues such as stress symptoms and performance anxiety were also common, with one-third of the youngest cohort reporting difficulties. Hearing-related problems appeared with notable frequency, with 17% reporting tinnitus and 14% measurable hearing loss: prevalence higher than in age-matched general populations. These findings highlight the importance of implementing preventive health strategies into youth music education.

Several prevention programs underscore the feasibility and potential benefits of early intervention. Türk-Espitalier et al. (2007) implemented daily warm-ups, cool-downs, and targeted workshops addressing posture, movement, and physical training with orchestral musicians from the ages of 14–24. The program was associated with reduced musculoskeletal strain, increased body awareness, and lower reported psychological barriers to seeking help, thus emphasizing the value of age-appropriate music physiology education. Music performance anxiety (MPA) also emerges early. Kenny and Osborne (2006) validated the Music Performance Anxiety Inventory for Adolescents (MPAI-A). Their results highlighted the need for the early identification and preventive intervetions of MPA in music education, showing its association with negative performance experiences and perfectionistic tendencies, that it peaks during mid-adolesence, and that it is higher in girls.

Additionally, hearing-related risks are well documented: based on an analysis of health insurance data from over three million German workers, Schink et al. (2014) reported significantly elevated hazard ratios (HRs) for professional musicians compared to the general population, including hearing loss (HR = 1.45, 95% CI [1.28, 1.65]) and tinnitus (HR = 1.57, 95% CI [1.34, 1.85]). The sound levels which musicians in an orchestra are exposed to often fall within ranges that are classified as potentially harmful to hearing (Neumann and Bork, 2011). This exposure depends heavily on the instrument being played, the seating position in the orchestra, the repertoire, and the venue (O’Brien et al., 2014).

The average sound levels measured during concerts range between 83.3 and 91.6 dB_A_ (Doswell Royster et al., 1991), and continuous exposure in classical symphony orchestras typically ranges between 85 and 95 dB_A_ depending on the instrument (Brusis, 2010; Neumann and Bork, 2011; Günther, 2015; Phillips and Mace, 2008). It is considered normal for both rehearsals and performances to significantly exceed 85 dB_A_—at which point hearing protection is recommended under occupational health guidelines—both in symphony orchestras (Neumann and Bork, 2011) and in conservatory orchestras (Richter et al., 2007). According to EU Directive 2003/10/EC (2003) (consolidated version as of July 26, 2019), the exposure limit for an 8-h working day is 87 dB_A_. As soon as 85 dB_A_ with a peak level of 137 dB_C_ is reached or exceeded, the employer is obliged to take measures to reduce noise exposure. The noise exposure in opera orchestras and orchestra pits is on average 3–5 dB higher than in symphony orchestras (Lee et al., 2005; Emmerich et al., 2007). Within the orchestra, the maximum level can reach up to 130 dB (Brusis, 2010; Williams and Stewart, 2011; Adrians, 2018), and in wind orchestras even up to 142 dB (Billeter and Hohmann, 2002; Penzkofer et al., 2015). The highest sound levels are typically measured near brass players, woodwind players, and drummers/percussionists (O’Brien et al., 2014). The noise exposure of orchestral musicians can be summarized as follows (see Table 1).

**Table 1:** Overview of noise exposure for orchestra musicians.

Musical instrument group	Typical average sound exposure	Details and maximum values	References
conductors	77.2–86.3 dB_A_	Lower sound exposure levels compared to other musical instruments	Richter et al. (2007) and Rodrigues et al. (2014)
String instruments	78.9–89.7 dB_A_ or 81–93 dB_A_	Upper strings: 86–93 dB_A_, Lower strings: 81–87 dB_A_	Doswell Royster et al. (1991), Woolford et al. (1988), and Rodrigues et al. (2014)
Woodwind instruments	86.2–97 dB_A_ or 90–94 dB_A_	The oboe reaches 95 to 102 dB when measured at the ear.	Woolford et al. (1988), Rodrigues et al. (2014), and Adrians (2018)
Brass instruments	83–94 dB_A_ or 87–97 dB_A_	Highest sound levels in the orchestra with more than 107 dB_A_, trumpet up to 93.7 dB_A_; French horn up to 96 dB_A_.	Doswell Royster et al. (1991), Woolford et al. (1988), Billeter and Hohmann (2002), Behar et al. (2008), and Toppila et al. (2011)
Percussion instruments	88–89.7 dB_A_ or upt to 98 dB_A_	Drums and timpani can briefly reach levels of up to 137 dB_A_.	Westmore and Eversden (1981), Toppila et al. (2011), and Rodrigues et al. (2014)

Sound exposure can be asymmetric depending on which side of the body the instrument is played: for violins and violas, the left ear is exposed to a higher level on average, with a difference of 3–8 dB (Brusis, 2010). During practice, an average of 93 dB_A_ was measured at the left ear and 86 dB_A_ at the right ear for violins and violas (Billeter and Hohmann, 2002). In contrast, higher sound levels are measured at the right ear for the harp and horn, with an average of 95 dB_A_ for the horn and 89 dB_A_ for the harp (both on the right) during practice (Billeter and Hohmann, 2002; Brusis, 2010). In addition to their participation in orchestras, musicians are also exposed to the sound from their individual practice (typically 10–15 h per week) and teaching (Brusis, 2010). Average levels between 60 and 107 dB have been measured during individual practice (O’Brien et al., 2014). Complementary studies by Pawlaczyk-Łuszczyńska and colleagues estimated lifetime hearing loss risk at 9–47% depending on instrument group, with brass, percussion, and high woodwinds being most affected. Excessive exposure to sound leads to noise-induced hearing loss in orchestra musicians, with damage occurring at 3 to 6 kHz (Doswell Royster et al., 1991; Richter et al., 2007; Jansen et al., 2009). In their study on 109 professional musicians from three major German orchestras, Emmerich et al. (2007) found permanent threshold shifts of more than 15 dB in over 50% of the participants. Their study showed that the risk of hearing damage increased with age, while surveys consistently show that fewer than 20% of musicians use hearing protection regularly, despite high awareness of the risks and the known practical limitations of currently available solutions. According to a recent study by Couth et al. (2021), there are a number of reasons why young professional musicians only use hearing protection sporadically: reduced control over the sound of their instrument, the occlusion effect (the instrument or the musicians voice seems to sound only in their head), intense performance pressure, lack of awareness of the problem, social “stigmatization,” uncomfortable fit, costs, or the fact that earplugs are easily get lost. Nevertheless, additional data from the Swiss National Accident Insurance Fund confirmed sustained exposure levels above 85 dB_A_ for many orchestral musicians, necessitating regular audiometric testing and mandatory hearing protection and thus complying with to occupational safety regulations (SUVA, 2020).

Beyond musculoskeletal and auditory health, music performance anxiety (MPA) represents another central challenge for young musicians. Virtual reality exposure training (VRET) enables practice under simulated evaluative conditions, and studies indicate that immersive VR may help alleviate anxiety and support performance-related skills (Williamon et al., 2014; Glowinski et al., 2015; Bissonnette et al., 2016). Recent systems combine stereoscopic 360° video with ambisonic audio (i.e., photorealistic, scene-captured environments, rather than avatar-based worlds) and, in some cases, real-time augmented acoustics (Frank et al., 2020; Bertsch and Frank, 2022). These adjustable sensory factors thus allow for the manipulation of perceived pressure from evaluators and the integration of psychophysiological monitoring (electrodermal activity, EMG, heart-rate–derived pulse, etc.) (Bertsch and Frank, 2022; Bertsch and Peschka, 2023). Preliminary evidence suggests that the body’s autonomic responses in VR mirror those of live-performance, indicating that VRET may provide an ecologically valid context for research and resilience training.

Building on this evidence, we introduce VRET.at: an open-access, free-to-use platform delivering photorealistic, venue-specific exposure scenarios (including major European concert halls). Unlike ad-hoc anxiety-provocation tasks, VRET.at offers standardized, curriculum-ready modules suitable for youth orchestras and music schools at low cost, lowering access barriers for institutions with limited resources. The platform natively interfaces with time-stamped event logs and physiological streams to enable event-locked analyses of “musically meaningful moments” (e.g., solo entries, errors, cues), thereby uniting training and measurement within a single scalable framework that complements established mental-skill and health-education programs (Williamon et al., 2014; Glowinski et al., 2015; Bissonnette et al., 2016; Bertsch and Peschka, 2023). By openly sharing core assets and implementation guidelines, VRET.at aims to facilitate broader adoption and replication, while providing a safe, ethically controllable environment to practice performing under pressure.

Emerging work combining continuous wearables with validated stress scales shows real-world feasibility (Ahmadi et al., 2025). Building on this, we implemented wearable stress monitoring devices, namely Empatica EmbracePlus in orchestral performance, to test the feasibility and ecological sensitivity of multimodal biomarkers and to link subjective experience with objective psychophysiological measures.

Against this background, the Erasmus+ project *The Future of Youth Orchestra – Addressing Physiological and Psychological Needs in Young Orchestral Musicians* (TFOYO; tfoyo.eu), which also serves as an open-access project platform hosting related interactive media, was launched as a transnational pilot initiative. The research was coordinated by the Austrian Society for Performing Arts Health & Music Psychology (ÖGfMM) in collaboration with the Norwegian National Youth Orchestra (NUSO) and the Landesjugendorchester Hamburg (LJO); TFOYO engaged over 130 musicians between the ages of 14–26 in two rehearsal phases that took place in Norway and Germany. Its overarching aims were to promote sustainable musician health, enhance resilience, and strengthen intercultural collaboration by implementing evidence-based training and digital tools into youth orchestra rehearsal culture.

The program integrated six health and training modules: (1) orchestral soundscape documentation, (2) practical interventions including daily warm-up and ergonomic training, (3) hearing protection education and audiometric testing, (4) Virtual Reality Exposure Training (VRET) aimed at addressing music performance anxiety, (5) wearable stress monitoring with smartwatches, and (6) quantitative surveys assessing physical and mental health. All materials were developed as open-access resources to ensure long-term availability for educators and orchestras. Beyond the scientific scope, the project aligned with the Erasmus+ priorities of health promotion, digital transformation, and youth participation, actively involving young musicians in committees and elements of project design. This participatory framework reflects the European Union’s emphasis on empowerment through co-creation and inclusive governance (European Commission, 2024, pp. 7–11). The project documentary can be found online (Borà and Rødevand, 2025).

## 2 Aims and research questions

The present study reports on a large-scale, multimodal field project conducted in real-world youth orchestra settings. Given the complexity of the intervention and the tightly constrained rehearsal context, the study follows a hierarchically structured set of aims and research questions.

The primary aim of this study was to develop, implement, and evaluate the feasibility and uptake of an integrated, multimodal health promotion framework for young orchestral musicians within intensive residential rehearsal camps. Rather than testing the efficacy of individual interventions under controlled conditions, the study focuses on whether such a comprehensive program can be logistically implemented into rehearsal culture, taken up by young musicians, and implemented without disrupting the artistic process.

In addition to this primary implementation-focused aim, the study pursued three secondary, exploratory aims:

1. to characterize acoustic, audiological, and psychophysiological exposure patterns of young musicians in real-world orchestral settings;
2. to explore short-term, pre–post changes in selected physical, psychological, and behavioral health indicators associated with participation in specific intervention modules;
3. to examine how individual differences in workload and stress-related behavior relate to health-relevant outcomes in youth orchestra contexts.

### 2.1 Primary research questions: feasibility and uptake (program implementation)

These research questions address the core focus of the study, namely the practical implementation of a multimodal, research-intensive health promotion program in youth orchestra rehearsal camps.

*RQ 3.1* (Feasibility): Is it logistically feasible to integrate a multimodal and research-intensive health program into the tightly scheduled daily routine of youth orchestra rehearsal camps without significantly disrupting the musical process?

*RQ 3.2* (Uptake): How do young musicians take up and engage with the various intervention modules (e.g., acoustic feedback, physical training, mental training), and which factors (e.g., instrument group, age) influence their willingness to participate in preventive measures such as hearing protection?

### 2.2 Secondary research questions I: acoustics and physiology (contextual health risks)

These research questions aim to objectively characterize health-relevant exposure conditions in youth orchestras. They provide essential contextual information for interpreting both feasibility outcomes and exploratory health indicators but are not designed to establish causal intervention effects.

*RQ 1.1* (Acoustics): What are the actual sound pressure levels and their spatial distribution during regular orchestra rehearsals, and to what extent do these levels exceed established thresholds for hearing risk in young musicians?

*RQ 1.2* (Audiology): Do young orchestra musicians already show signs of hearing-related vulnerability (e.g., high-frequency threshold shifts) despite their age, and do such indicators differ between instrument groups?

*RQ 1.3* (physiology): Can physiological parameters (e.g., skin conductance, heart rate variability), collected via wearable technology, be used to capture individual and context-specific stress responses during rehearsals and performances, and can these responses be temporally related to specific musical passages?

### 2.3 Secondary research questions II: psychology and behavior (exploratory intervention-related outcomes)

These research questions explore short-term, exploratory associations between participation in specific intervention modules and selected psychological, physical, and behavioral indicators. Given the non-controlled design, these questions are explicitly formulated as exploratory and do not permit confirmatory inference.

*RQ 2.1* (Mental Health): Is participation in combined psychological interventions (e.g., mental training and virtual reality exposure training for performance anxiety) associated with short-term changes in music performance anxiety?

*RQ 2.2* (physical health): Is participation in integrated physical training modules (e.g., BodyFit sessions and structured warm-ups) associated with changes in musicians’ ergonomic awareness and self-reported intensity or prevalence of performance-related musculoskeletal complaints in highly stressed body regions?

*RQ 2.3* (Behavior): How are individual stress management styles related to rehearsal behavior (e.g., dysfunctional practice patterns) and to indicators of time investment and workload, conceptualized here as “musical busyness” (see 4.8.4)?

Taken together, the research questions follow a multilevel evaluation logic, with feasibility and uptake constituting the primary evaluative focus of the study. Acoustic, audiological, and physiological measures provide essential contextual information on exposure conditions, while psychological, physical, and behavioral indicators are examined exploratorily to inform future, more controlled intervention research.

## 3 Methods

### 3.1 Study design and participants

**Figure 1:**
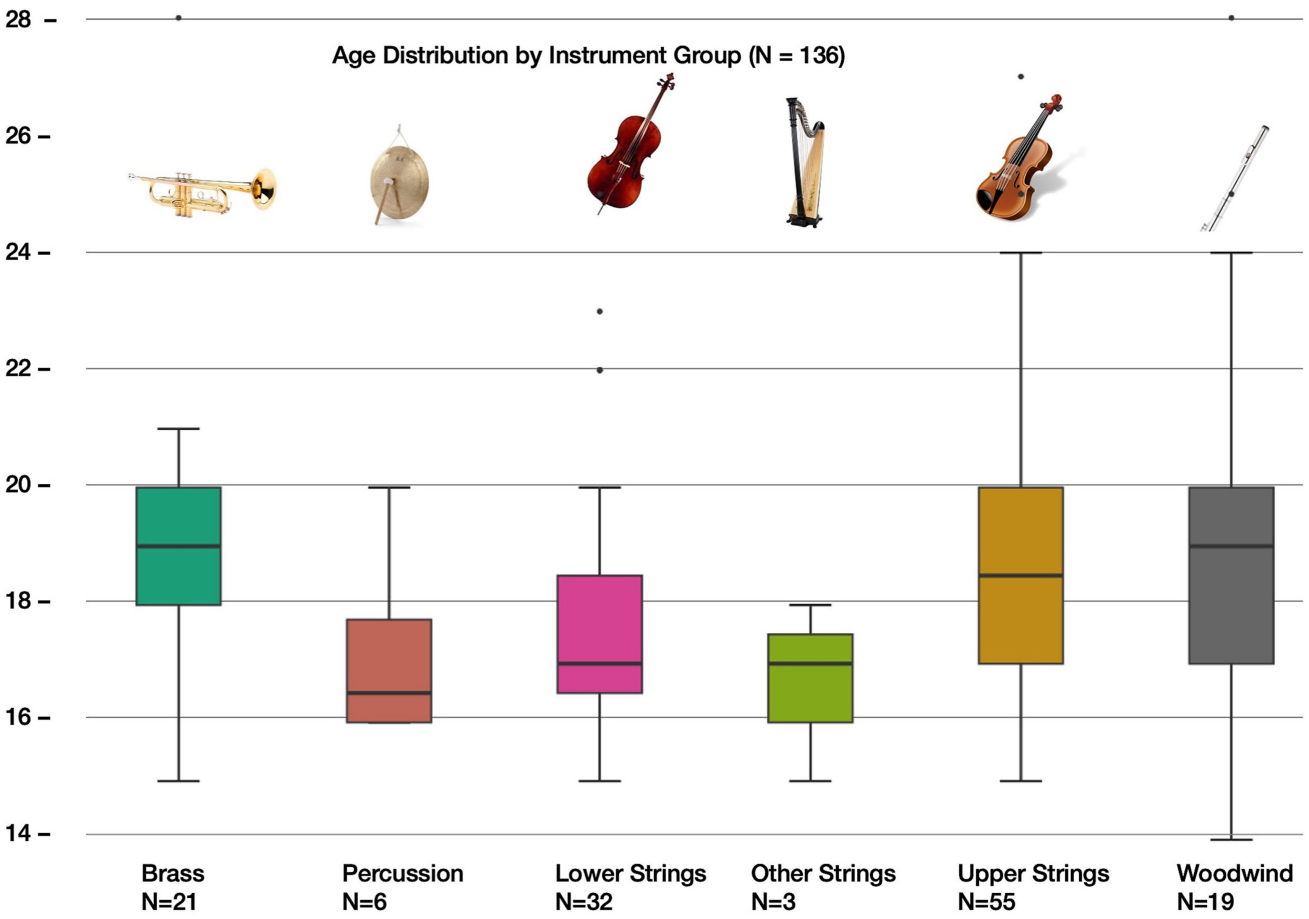
Age distribution across instrument groups in the youth orchestras.

A total of 136 musicians from two European youth orchestras participated: the Landesjugendorchester Hamburg (LJO; *n* = 67, 49%) and the Norwegian National Youth Orchestra (NUSO; *n* = 69, 51%). The cohort covered all major orchestral sections: upper strings (*n* = 55), lower strings (*n* = 32), brass (*n* = 21), woodwinds (*n* = 19), percussion (*n* = 6), and others such as keyboard and harp (*n* = 3). Ages ranged from 13 to 27 years (M = 18.3); 48% were adults, 50% minors, and 2% undeclared. Gender distribution was balanced across the sample. The age distribution across instrument groups is shown in Figure 1, indicating slightly higher mean ages among wind and upper string players. Participation was voluntary; written informed consent was obtained from all adult participants, and from legal guardians for participants under 18 years of age.

**Figure 2:**
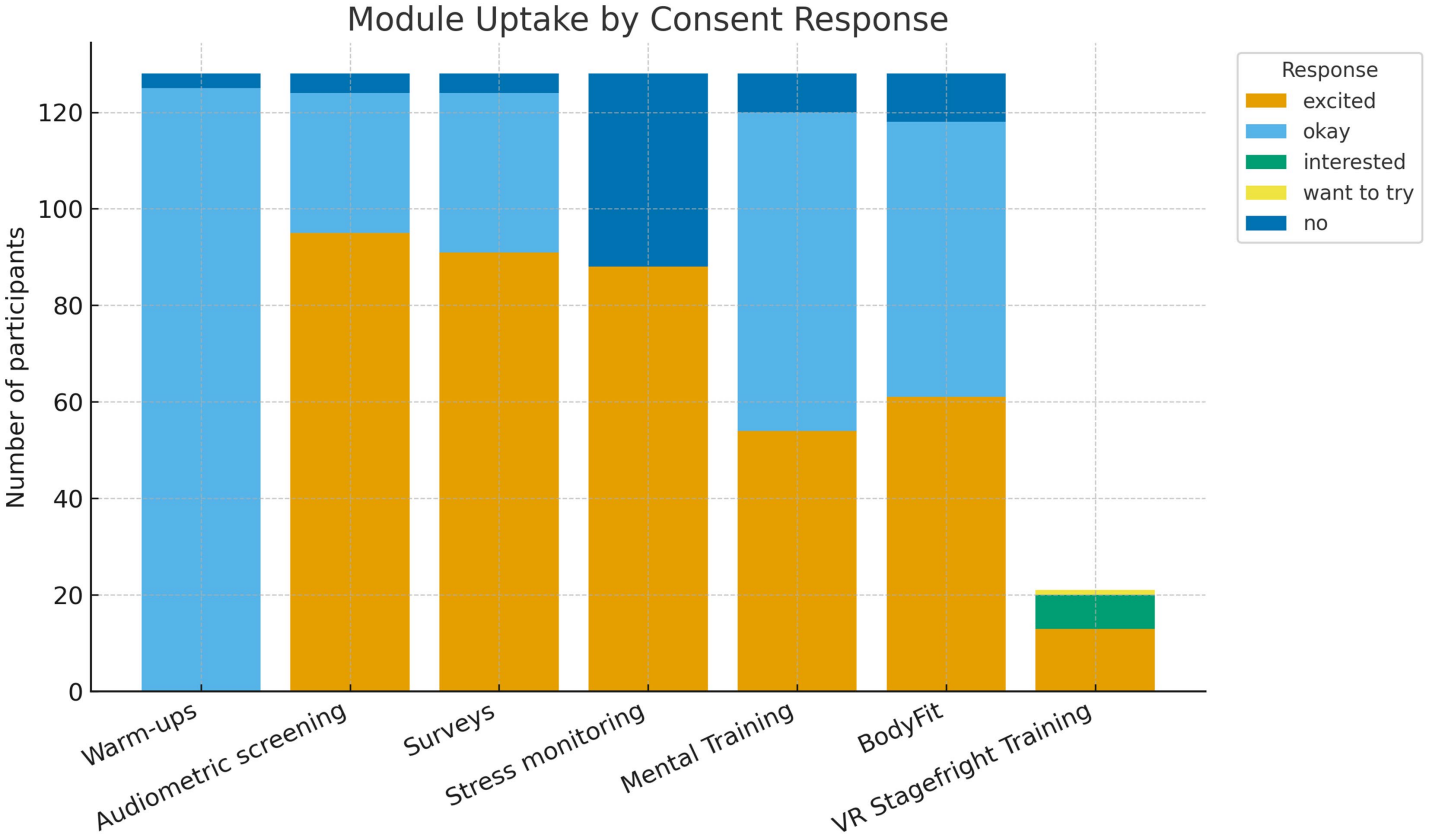
Uptake of intervention modules by consent response. Stacked bars show the number of participants per module categorized as “excited,” “okay,” “interested,” “want to try,” and “no.” Data are based on pre-camp consent forms (*N* = 136).

All musicians participated in the core program, which was composed of daily orchestra warm-ups, hearing protection lessons, and baseline health screenings. Additional intervention modules were optional and selected by the participants in advance through pre-camp consent forms, either filled out by the participants themselves or, in cases of underage participants, by their parents. Uptake was high across modules; for example, 61 participants (47.7%) marked “I would love to do this” for BodyFit, and 54 (42.2%) did so for Mental Training (assigned the label “excited” in Figure 2). Most of the remaining respondents selected “It is okay to be assigned to this group” (see Figure 2).

Due to the nature of the project, methodological considerations regarding sample size for the overall questionnaires were considered superfluous, since the number of attendees was determined by the program’s organizational capacity. The musicians therefore functioned as a convenience sample.

Regarding the intervention groups, we aimed to assign around 20 participants to each intervention group to be able to test for middle to large effect sizes (16–20 people for Pearson’s r as well as Cohen’s d > 0.5), taking a natural drop-out risk of 5–10% into account. As funding was limited to only 15 smartwatches, only large effects were able to be accounted.

Final groups were allocated based on participants’ preferences and included 57 musicians in BodyFit (27 adults, 30 minors), 54 in Mental Training (28 adults, 26 minors), and 13 in VRET. Overall, fewer than 8% of participants declined participation in any additional module.

### 3.2 Camps and project structure

The project consisted of two main phases. The first phase was a 10-day summer camp in Bodø from August 3–11, 2024, which culminated in a concert at the Stormen Concert Hall. The second phase was a 3-day autumn camp in Hamburg from October 2–4, 2024, culminating in a public concert under professional performance conditions at the Elbphilharmonie on October 5, 2024. This design enabled repeated measurements in intensive rehearsal and high-pressure performance contexts.

A brief feedback questionnaire was administered immediately after the Bodø phase, followed by a comprehensive questionnaire six weeks later to enable comparative analyses. All participants took part in daily collective interventions (tutti warm-up routines, the “Get in the Zone” instructional video, an educational lecture format, and audiometric screening) and were additionally assigned to one intervention track comprising three structured sessions of approximately 60 min per week.

The orchestral camp in Bodø, Norway, followed an intensive daily schedule of tutti and sectional rehearsals, health-related workshops, and intervention sessions. Short breaks and frequent transfers between accommodations, rehearsal venues, and dining facilities affected physical strain. Evening activities such as concerts and social events further reduced recovery opportunities. The Arctic summer light conditions may have influenced sleep and recovery patterns.

The joint concert program featured György Ligeti’s Lontano, excerpts from Sergei Prokofiev’s Romeo and Juliet Suites Nos. 1 and 2, and Ottorino Respighi’s Pini di Roma, conducted by Torodd Wigum and Johannes Witt. In Bodø, the program additionally included Ingebjørg Vilhelmsen’s Festivity (Fest Overture).

The study was embedded within the Erasmus+ pilot project *The Future of Youth Orchestra – Addressing Physiological and Psychological Needs in Young Orchestral Musicians (TFOYO)*, coordinated by the Norwegian National Youth Orchestra (NUSO) in collaboration with the Landesjugendorchester Hamburg (LJO) and the Austrian Society for Performing Arts Health & Music Psychology (ÖGfMM), with the scientific conception, study design, and evaluation led by the ÖGfMM research group. A mixed-methods approach combined quantitative measures (e.g., audiometric thresholds, SPL exposure, biometric stress markers) with qualitative data (e.g., self-reported anxiety and reflections on training experiences) to support a comprehensive evaluation of the program. Rather than testing isolated components, quantitative and qualitative data were jointly interpreted to assess the feasibility and acceptance of the overall multimodal intervention framework. The overarching aim was to support health awareness, resilience, and performance readiness in young musicians by integrating evidence-based interventions into youth orchestra rehearsal culture.

### 3.3 Acoustic soundscape analysis and mapping

To document the acoustic conditions within the orchestra, sixteen measuring microphones (Behringer ECM8000) recorded in a multitrack session in Adobe Audition (16 mono tracks, each 48.000 Hz, 32 Bit) were placed at representative musician positions during a rehearsal of Ingebjørg Vilhelmsen’s orchestral piece, “Festivity” (Vilhelmsen, 2023). This piece was chosen because, within three minutes, it runs through a variety of volumes between *ppp* and *fff*, which are the typical dynamic extremes in an orchestra. Calibrated to 114 dB_SPL_ at 1000 Hz using an ND9 acoustic calibrator, the microphones were placed at ear level for musicians from 15 instrument groups and the conductor. This allowed the musicians’ sound exposure during the performance to be recorded as sound pressure levels in dB_SPL_. In addition, these sixteen synchronized orchestra tracks were used to create a soundscape visualization in which the listener can interactively observe the listening positions of individual orchestral musicians to explore the orchestra’s internal soundscape. Additional documentary recordings were made using an Insta360 Pro camera with 360° Ambisonic audio and 8 K video capabilities, providing immersive audiovisual documentation.

### 3.4 Audiometric screening

Audiometric testing was conducted by a certified occupational health physician using a Zeisberg CA350 clinical USB audiometer with AudioApp software and calibrated HDA280 headphones. Standard air-conduction thresholds (125–8,000 Hz) were assessed following pure-tone audiometry protocols. In total, 77 participants (41 female, 36 males; aged 14–27 years) completed audiometry during the Bodø rehearsal phase, covering all major instrumental groups (50 strings, 11 woodwinds, 16 brass, 1 percussion). A small number of staff musicians and instructors (up to 57 years old) were also included to provide reference data. As part of the hearing-health module, all musicians received an illustrated fact sheet on safe exposure limits and hearing-protection strategies by Bertsch et al. (2024), followed by an interactive Kahoot^®^ quiz attended by over 100 participants. This combined diagnostic-educational approach supported awareness and prevention. If no clinical audiometer is available in follow-up studies or for self-diagnosis by musicians, the BHI Quick Hearing Check (Kochkin and Bentler, 2010) can be recommended as an inexpensive and time-saving alternative for evaluating hearing thresholds.

### 3.5 Psychophysiological stress monitoring

Fifteen musicians (10 string players, 5 wind players) participated in continuous psychophysiological monitoring using Empatica EmbracePlus smartwatches. Participation was voluntary and restricted to musicians aged 18 years or older who had expressed strong interest in this module during pre-camp consent procedures. All participants were members of the Hamburg orchestra cohort, as devices were distributed and configured locally in Hamburg for logistical reasons.

The smartwatches recorded electrodermal activity (EDA; tonic skin conductance level in μS, 4 Hz), blood volume pulse (BVP; 64 Hz), wrist skin temperature (1 Hz), and tri-axial accelerometry (64 Hz). In the present study, analyses focused on tonic EDA, pulse rate derived from BVP, and wrist temperature. Other parameters provided by the device (e.g., HRV, sleep detection) were not included in the analyses reported here.

Physiological data were time-stamped and transmitted via dedicated smartphones to a GDPR-compliant secure cloud infrastructure, where all data were stored under anonymized numeric participant IDs. Only the participants themselves and the project lead could link IDs to individual identities, in accordance with the approved ethics protocol.

To enable event-related analyses, physiological data streams were temporally synchronized with private video recordings of rehearsals, concerts, and virtual reality sessions using shared timecodes. Musical events (e.g., entrances, solos, structurally demanding passages) were identified based on annotated scores and video footage, allowing alignment of physiological signals at the level of minutes and, where applicable, seconds.

Qualitative self-reports were collected through audio-recorded semi-structured interviews comprised of open questions conducted before and after concerts, which were subsequently transcribed. In addition, follow-up questions were sent by e-mail approximately one week after the project, asking participants to describe particularly memorable or emotionally salient moments during the concert. All qualitative data were linked to the corresponding physiological recordings via the anonymized participant IDs and used to support multimodal interpretation.

Analyses were exploratory and focused on assessing the feasibility and ecological suitability of wearable stress monitoring in live orchestral performance contexts, including the robustness of data acquisition, the presence of movement artefacts, and the interpretability of individual time courses.

### 3.6 Psychological health: virtual reality exposure training (VRET)

The virtual reality exposure training (VRET) module was designed to simulate high-stakes performance situations in photorealistic concert venues, with the primary aim of examining the feasibility and ecological suitability of VR-based exposure settings within youth orchestra rehearsal camps. Visual recordings were captured using stereoscopic 8 K 360° images and videos from established concert venues, including the Elbphilharmonie in Hamburg and concert stages in Vienna. The performance scenarios additionally included a big band solo rehearsal setting. Although the Augmented Practice Room system (developed by IEM Graz and implemented at the Motion-Emotion Lab, mdw Vienna) allows real-time augmented room acoustics, it was not sufficiently portable for use during the Bodø camp. Accordingly, the VRET module employed pre-rendered visual scenes without real-time acoustic augmentation.

Participation in the VRET module was voluntary and restricted to brass players, as this instrument group can perform without direct visual reference to the instrument, ensuring methodological comparability across participants. Following the consent procedure, 15 wind players expressed strong interest in participating. Thirteen participants (aged 16–20 years) attended an initial introductory and briefing session and thus constituted the VRET sample (*N* = 13). Across the camp, six VRET sessions of approximately 60 min were offered; participants were invited to select up to three sessions depending on availability. Individual active playing time within each session was approximately 10–15 min, while other participants observed the session in a group setting.

During the Bodø camp, VRET sessions were conducted in a classroom environment using Meta Quest Pro head-mounted displays. To minimize the risk of cybersickness, all scenarios employed fixed panoramic viewpoints without artificial spatial movement. The visual scenarios were comprised of solo audition excerpts, selected orchestral passages, and improvisation prompts. Stress-inducing elements were introduced through simulated jury presence and camera recording, accompanied by task instructions (e.g., performing short, memorized excerpts), while no explicit time pressure was imposed. All sessions were video-recorded with second-level timecodes; selected performances were additionally live-streamed via a private YouTube channel to increase evaluative salience. Participants were informed about the recording procedures and were free to discontinue participation at any time in accordance with the approved ethics protocol. No cases of cybersickness were reported.

The VRET module was designed for multimodal research integration. Physiological monitoring via Empatica EmbracePlus smartwatches was conducted for a subset of participants who also took part in the stress-monitoring module (see Section 3.4). In addition, time-stamped VR event information (e.g., task onset, performance phases) and brief qualitative self-reports collected after sessions were used to enable exploratory, case-based, event-locked analyses linking task characteristics with psychophysiological responses. The focus of the VRET module was on feasibility, technical robustness, and the interpretability of synchronized multimodal data in live rehearsal and performance contexts rather than on evaluating therapeutic efficacy.

### 3.7 Physical health and behavior: practical interventions

The practical intervention program consisted of daily warm-ups, instructional media, educational activities, and small-group workshops, all delivered by certified specialists and integrated into the rehearsal schedule.

#### 3.7.1 Daily warm-ups and mental preparation

At the beginning of the morning tutti rehearsals and dress rehearsals, the full orchestra participated in ~10-min warm-up sessions designed to reduce musculoskeletal strain and enhance focus. Framed as the first 10 min of rehearsal (not a pre-rehearsal extra), the routine achieved near-universal uptake and punctual participation across sections. Sessions combined (a) *physiological warm-ups* (low-impact mobility, posture, and circulation exercises) and (b) *mental warm-ups* (breathing, relaxation, and confidence-building techniques).

#### 3.7.2 Instructional video “Get in the Zone”

A four-minute instructional video featuring eight guided exercises (balance, focusing, spinal mobility, flexibility, facial relaxation, sensory activation, tension release, dynamic shaking, and power posing) was produced and used during the camps as a standardized collective warm-up resource. The video, titled “Get in the Zone,” was developed collaboratively by three certified experts in music physiology, mental training, and performing arts medicine, and integrates established, evidence-informed exercises commonly used in musician health and performance preparation. It was published under a CC BY-NC 4.0 license for open-access dissemination (Österreichische Gesellschaft für Musik und Medizin (ÖGfMM) Primafit, 2024).

#### 3.7.3 Health education

A dedicated evening session (“Kahoot Fun Night”) combined illustrated fact sheets on safe sound exposure with interactive lectures and a game-based quiz format to reinforce hearing-health, physical and mental well-being, and stress-prevention strategies.

#### 3.7.4 Group-based workshops

On the consent/registration form, musicians indicated their openness and preference for the afternoon workshops (response options included “excited” and “okay”). To ensure balance, participants who were open to both BodyFit and Mental Training were randomly assigned, stratified by age, gender, and instrument group. When a clear preference was expressed (e.g., excited for one and okay for the other), it was honored in up to ~30% of cases to accommodate participant autonomy while maintaining overall balance. The workshops included the following:

- *Mental training* (Groups A/B): breathing techniques, progressive muscle relaxation, mental practice strategies, and self-confidence-boosting methods. Group A: minors (<18). Group B: adults (≥18)
- *BodyFit for young orchestra musicians* (Groups C/D): body-awareness, compensatory exercises, and instrument-specific ergonomics. Group C: minors (<18). Group D: adults (≥18). The BodyFit workshops were informed by established principles of music physiology and performing arts medicine, drawing on long-standing practice-based knowledge rather than a single standardized protocol.
- *Virtual reality exposure training (VRET)* (Group E, limited to wind/brass): all eligible players expressed high interest and were assigned accordingly. VRET used immersive 360° audiovisual simulations of performance environments to familiarize musicians with high-pressure situations.

Workshop attendance was logged; spontaneous participation outside assigned groups was noted. All sessions were scheduled around the rehearsal plan without interfering with artistic preparation.

Alongside live interventions, the program included digital handouts, instructional media, and online reports to ensure sustained accessibility for educators and orchestras beyond the camp phases.

### 3.8 Surveys and test inventories

To assess psychological, physical, and hearing-related health parameters, a standardized survey battery was administered at two measurement points: pre-camp (July 2024, before the Bodø phase) and post-camp (September/October 2024, before the Elbphilharmonie concert).

#### 3.8.1 Instruments

- Music Performance Anxiety Inventory for Adolescents (MPAI-A; Osborne and Kenny, 2005): assesses cognitive, physiological, and behavioral dimensions of performance anxiety.
- Performance Anxiety Questionnaire (adapted K-MPAI, Kenny, 2011): extended screening of music performance anxiety (40- and 15-item versions).
- Stressverarbeitungsfragebogen (SVF-78; Erdmann and Janke, 2008): evaluates coping strategies for stress, distinguishing between functional and dysfunctional patterns.
- Leistungsmotivations-Inventar (LMI; Schuler et al., 2001): measures achievement motivation (goal setting, persistence, self-confidence).
- Dysfunctional Practice Behavior Scale (Roos et al., 2017): identifies harmful practicing habits, such as neglecting own physical and mental health in favor of practicing.
- Hearing Health Questionnaire (custom): assesses perceived hearing problems (e.g., tinnitus, hyperacusis), awareness of acoustic risks, and hearing-protection behavior.
- Musicians’ Health Items [adapted from Samsel et al. (2005) and Gembris and Ebinger (2014)]: addresses posture, muscle tension, pain occurrence and coping with pain, body awareness, health knowledge, and professional aspirations.
- Musical Stress Coping Questionnaire (custom): additional items on practice-related strain, adaptive vs. maladaptive strategies, and daily stressors.

#### 3.8.2 Administration

- Surveys were administered online (SoSci Survey) in German and English.
- Participation was voluntary; items could be skipped.
- Participants completed the online surveys independently at home at two time points: four weeks prior to the Bodø Camp and four weeks before the Hamburg Camp. They were informed and reminded via email to ensure timely participation.
- The full in pre-camp survey battery required approximately 55 min to complete.
- Demographic and musical background data were also collected (instrument, years of training, lesson history, ensemble activity, weekly rehearsal frequency, and concert experience).
- Those inventories which were not available in a standardized version in English, we translated together with native speakers in the field and let target group-equivalent people check for possible misunderstandings.

### 3.9 Data handling

Each participant received a pseudonymized study ID to link pre- and post-camp data while preserving confidentiality. Data were stored in a secure, password-protected database in accordance with the ethics approval granted by the University of Music and Performing Arts Vienna (May 2024).

## 4 Results

### 4.1 Acoustic soundscape analysis and mapping

By summarizing the dynamic values at the ears of the individual musicians (see Tables 2, 3), it can be seen, particularly at the median and maximum levels, that the ears of the conductor (79.0 dB_SPL median_, 91.8 dB_SPL max,_ 75.2 dB_A median_, 83.8 dB_A max_) and double bass players (79.8 dB_SPL median_, 90.8 dB_SPL max,_ 73.5 dB_A median_, 83.0 dB_A max_) are at least at risk. In contrast, the ears of wind and percussion instrumentalists are particularly at risk: with median levels of up to 87.8 dB_SPL median_ (83.0 dB_A_, French horn) and maximum values of up to 101.1 dB (95.5 dB_A_. timpani) they mainly play in a range above 85 dB_SPL_, where sound begins to have a harmful effect on hearing after extended exposure.

**Table 2:** Sound pressure level exposure (in dB_SPL_) of the conductor and musicians playing string instruments (top) as well as wind and percussion instruments (bottom), measured at ear level within the respective instrument group.

Instruments	Conductor	Violin 1, front	Violin 1, rear	Violin 2, front	Violin 2, rear	Viola	Violon-cello	Double Bass
Mean	77.52	80.74	78.94	80.51	80.65	80.78	79.80	77.58
Median	78.96	82.62	80.53	82.23	82.81	82.83	82.01	79.79
q1 (25%)	73.77	78.28	75.83	77.43	76.95	75.25	74.34	72.68
q3 (75%)	83.38	85.94	84.91	86.60	86.42	88.00	87.04	84.58
IQR	9.61	7.66	9.08	9.17	9.47	12.75	12.70	11.90
Min	39.88	41.13	42.09	42.14	43.53	43.85	41.01	39.70
Max	91.81	94.01	91.99	94.28	95.83	96.13	94.01	90.79
Range	51.93	52.88	49.90	52.14	52.30	52.28	53.00	51.09

Instruments	Flute clarinet	Bassoon	French horn	Trumpet	Trombone	Tuba	Timpani	Percussion
Mean	82.06	84.05	84.48	81.86	81.85	81.08	82.86	78.40
Median	85.34	86.84	87.78	84.27	85.45	84.99	84.39	80.48
q1 (25%)	77.08	78.48	83.06	78.02	74.47	73.62	76.30	73.08
q3 (75%)	88.88	91.98	90.90	88.76	90.14	89.37	90.73	85.61
IQR	11.80	13.50	7.84	10.74	15.67	15.75	14.44	12.54
Min	38.90	40.33	39.33	37.65	40.41	40.03	41.12	39.86
Max	93.71	97.45	97.45	94.86	95.85	94.57	101.09	96.21
Range	54.81	57.12	58.12	57.21	55.44	54.54	59.97	56.35

**Table 3:** Sound pressure level exposure (in dB_A_) of the conductor and musicians playing string instruments (top) as well as wind and percussion instruments (bottom), measured at ear level within the respective instrument group.

Instruments	Conductor	Violin 1, front	Violin 1, rear	Violin 2, front	Violin 2, rear	Viola	Violon-cello	Double Bass
Mean	74.2	77.9	76.1	76.8	78.5	77.0	74.9	72.4
Median	75.2	79.6	77.3	78.1	79.8	78.2	75.9	73.5
q1 (25%)	70.81	75.08	72.88	73.99	75.08	72.81	70.85	68.36
q3 (75%)	78.22	82.81	81.12	81.74	83.9	82.63	80,23	77.88
IQR	7.41	7.73	8.24	7.75	8.82	9.82	9.38	9.52
Min	20.9	20.9	20.9	20.9	19.2	20.7	20.6	18.6
Max	83.8	90.1	85–7	84.7	87.7	95.3	83.1	83.0
Range	62.2	69.2	64.9	63.9	68.5	65.2	62.5	64.3

Instruments	Flute clarinet	Bassoon	French horn	Trumpet	Trombone	Tuba	Timpani	Percussion
Mean	79.6	81.1	81.0	79.0	77.7	75.2	77.3	73.9
Median	81.3	82.3	83.0	80.5	79.0	76.4	78.5	75.1
q1 (25%)	75.02	75.28	76.94	73.47	71.25	69.53	72.03	68.87
q3 (75%)	86.09	88.73	87.62	86.61	85.58	82.0	83.52	80.19
IQR	11.07	13.45	10.68	13.14	14.33	12.47	11.49	11.32
Min	23.3	26.8	28.1	24.2	26.2	26.2	26.7	26.8
Max	90.1	94.5	93.1	91.1	91.4	87.1	95.5	88.4
Range	66.8	67.7	65.1	66.9	65.3	60.9	68.9	61.6

**Figure 3:**
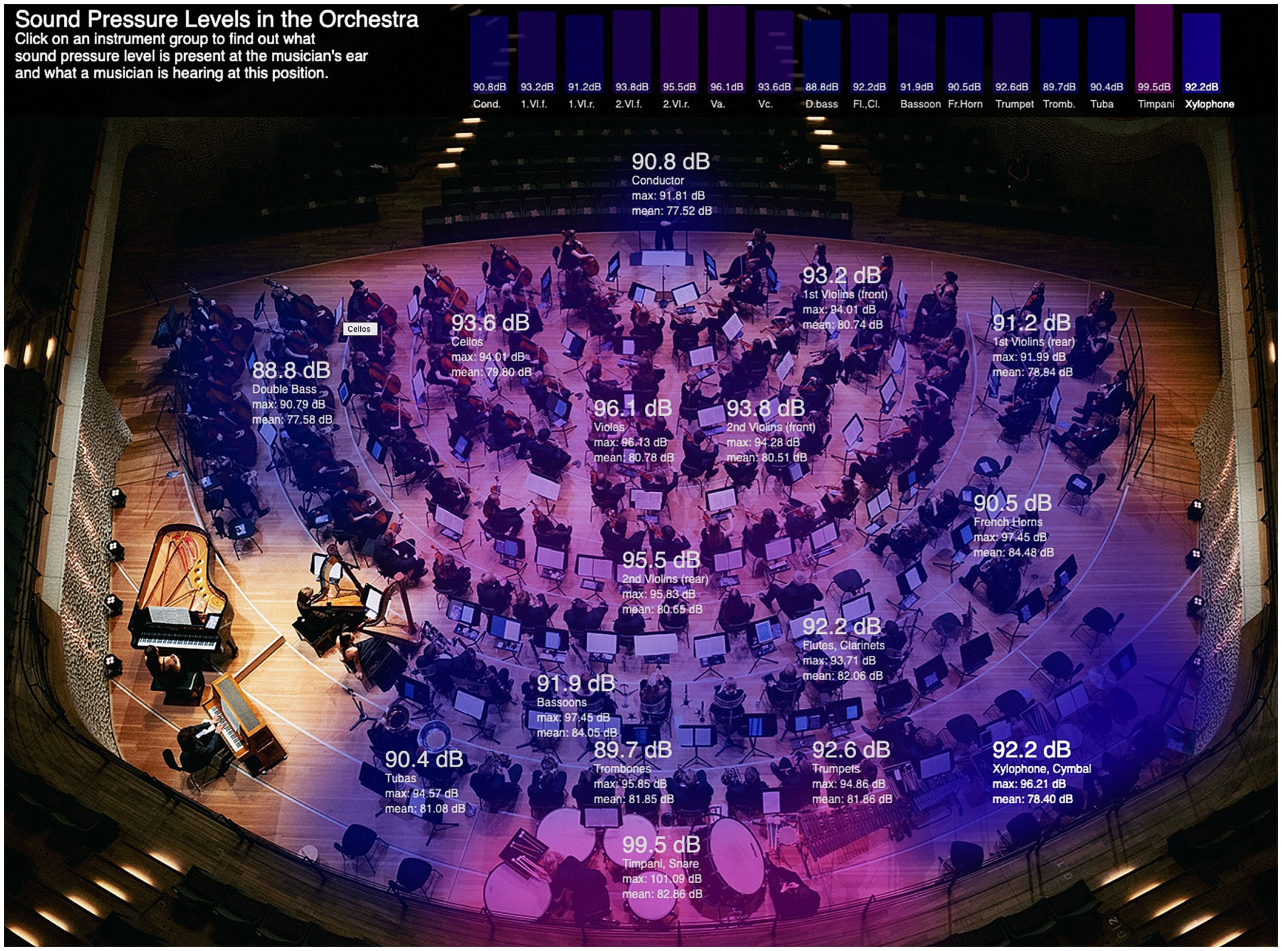
Screenshot of an interactive sound pressure level heatmap of a symphony orchestra at: https://muwiserver.univie.ac.at/tfoyo/. Colors ranging from green (35 dB) to violet (over 100 dB) indicate the sound level of the individual instrument groups. By clicking on the instrument groups, you can acoustically place yourself in the position of the respective orchestra musician during the piece [the Norwegian National Youth Orchestra (NUSO) together with the Landesjugendorchester Hamburg (LJO) plays Ingebjørg Vilhelmsen’s orchestral piece, “Festivity”] (Vilhelmsen, 2023).

As shown in Figure 3, the spatial distribution of sound pressure levels within the orchestra is visualized online^1^ as an interactive heatmap based on the recordings of the 16 calibrated microphone positions. Color gradients represent the sound pressure level measured at each musician’s ear, ranging from 37 to more than 100 dB_SPL_, with the highest exposures observed among brass and percussion players. The visualization highlights the acoustic heterogeneity across orchestral sections and enables users to listen from each instrument’s perspective, comparing perceived timbre and intensity across positions. Furthermore, a virtual rehearsal tour created with 3DVista Virtual Tours integrates audio samples and navigable viewpoints, offering an additional educational resource on orchestral acoustics^2^.

### 4.2 Audiometric screening

Audiometric screening (125–8,000 Hz) revealed that most tested musicians exhibited hearing thresholds within normal limits. However, early high-frequency threshold shifts (>20 dB_HL_ above 4 kHz) were observed in several brass players, particularly trumpets, horns, and trombones. In some cases, these shifts were asymmetric between ears, consistent with the directional exposure pattern of the brass instruments.

Group-level observations:

- Strings (*n* = 50): Largely normal hearing thresholds, with occasional mild hearing loss at 6 and 8 kHz.
- Woodwinds (*n* = 11): Predominantly normal audiograms, with isolated mild high-frequency notches (e.g., clarinet, oboe).
- Brass (*n* = 16): Higher prevalence of early high-frequency threshold shifts, in some cases asymmetric.
- Percussion (*n* = 1): No measurable deficits; thresholds were exceptionally good, likely due to consistent use of customized hearing protection.

Overall, most young musicians in this cohort still showed healthy auditory profiles, yet the earliest signs of high-frequency hearing decline were already evident in high-exposure groups. These findings underscore the importance of early preventive education and action.

**Figure 4:**
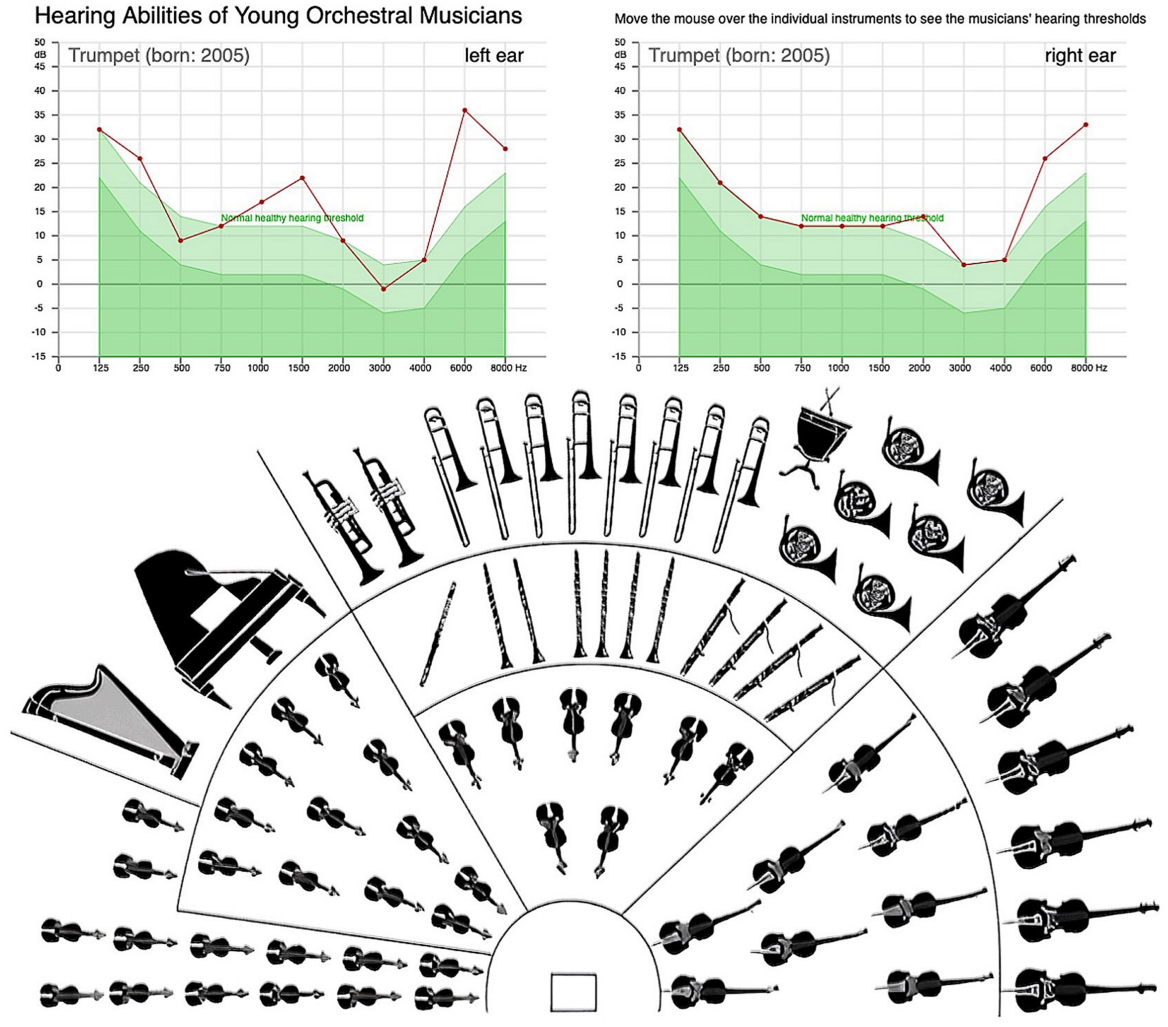
Screenshot of interactive audiometric profiles of 77 young orchestral musicians at: https://muwiserver.univie.ac.at/tfoyo/audiometrie, selectable via mouseover. The graphs show representative audiograms from a trumpet player, illustrating individual left and right ear thresholds compared to the normative healthy hearing range (light green area).

As shown in Figure 4, audiometric results of the youth orchestra musicians demonstrate that most hearing thresholds fall within the normal range (light green area). However, early high-frequency threshold shifts are visible in several brass players, particularly trumpets, horns, and trombones, indicating initial signs of exposure-related hearing loss. The interactive online audiogram tool^3^ allows users to explore hearing thresholds for each instrument group and compare left- and right-ear measurements across the orchestra.

To address these risks, all participants received a “Hearing Basics” fact sheet (Bertsch et al., 2024) and joined an educational session on acoustic safety, including a Kahoot^®^ quiz attended by nearly 100 musicians. Qualitative feedback indicated that the session increased awareness and motivation to use hearing protection more consistently.

### 4.3 Physiological stress monitoring

#### 4.3.1 Group-level patterns

We monitored 15 musicians with EmbracePlus smartwatches across rehearsal and concert days, recording electrodermal activity (EDA), pulse rate, wrist temperature, accelerometry, activity counts, and steps, as well as respiration and heart-rate variability (HRV; both derived from blood-volume pulse, BVP) during resting phases only. All signals were processed offline and synchronized second-by-second with annotated scores and time-coded video recordings to enable context-aware, event-related interpretation (Bertsch, 2024).

**Figure 5:**
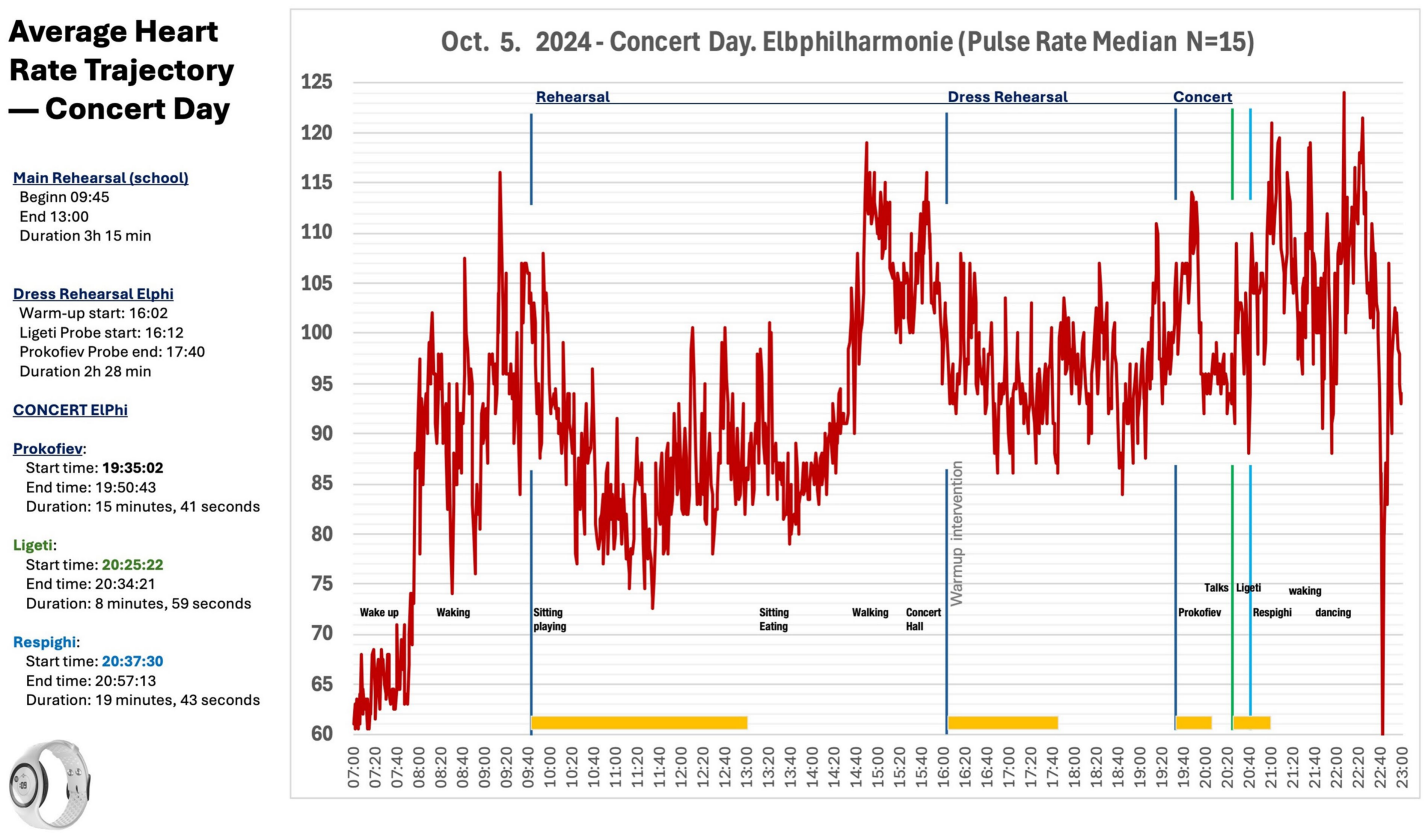
Median heart-rate trajectory on the concert day (Elbphilharmonie, October 5, 2024; *N* = 15). The time-aligned group-median pulse illustrates elevated values during morning travel and arrival (>110 bpm), a reduction during the orchestra warm-up (~95 bpm), and renewed elevations upon entering the hall and at concert onset. A transient decrease is visible during the opening speech, followed by increases during Ligeti and Respighi. The highest median values occur during applause and post-concert social interaction. Vertical markers denote rehearsal, dress rehearsal (warm-up ~16:00), and concert segments.

On the concert day in Hamburg, the main rehearsal took place at a school (09:45–13:00). As shown in Figure 5, the group-median heart rate exceeded 105 bpm during morning travel and arrival, before gradually settling during the rehearsal period. Around 14:40, upon arrival at the Elbphilharmonie and first exposure to the concert hall environment and stage view, the median group level rose again to approximately 115 bpm, consistent with anticipatory activation. At 16:02, during the brief on-stage warm-up and mental focusing phase that opened the dress rehearsal on the main stage (16:02–16:12), the median declined to approximately 95 bpm and remained comparatively stable. Heart-rate values increased again during hall entry and concert onset with Prokofiev. The values declined during the extended opening speech, and rose throughout the subsequent program, with pronounced elevations during Ligeti and Respighi. The highest group-median levels of the day were observed during the final applause and post-concert social interaction, consistent with heightened positive arousal. These patterns are descriptive and observational, indicating temporal associations with situational and musical events rather than causal effects; individual trajectories varied around the group median.

#### 4.3.2 Participant-reported experiences during wearable stress monitoring

Self-reports collected alongside the wearable recordings indicated high perceived realism of the monitoring procedure and helped identify situation-specific stressors and modulators of arousal. Several participants described anticipatory activation before salient musical events or exposed entries (e.g., initial tones in Lontano or offstage trumpet cues in Pini di Roma), whereas others emphasized logistical or contextual stressors, such as delayed meals, transport pressure, or performing from elevated positions, which coincided with physiological arousal outside strictly musical peaks.

Instrument-specific demands further shaped perceived load profiles. Upper string players frequently referred to fast Presto passages and high counting demands in Lontano; low strings and violas reported sustained-tone fatigue and back or arm strain; brass players highlighted embouchure fatigue and concerns regarding accuracy in the high register. Many participants reported only mild nervousness during rehearsals but markedly increased tension immediately before concert onset, followed by a rapid reduction in arousal after initial entries. A subset of participants wore the device on the bowing (right) hand, which plausibly accounts for movement-related artefacts during vigorous passages, as noted in the data-quality assessment. Despite this, these participants generally perceived the recordings as reflective of their subjective effort and arousal.

Taken together, these contextualized self-reports align with the event-locked physiological patterns observed during both concert performance and virtual reality exposure sessions, supporting the interpretability of the time-synchronized wearable data and illustrating how musical structure, performance context, and logistical factors may jointly contribute to sympathetic activation (anonymized participant interviews, TFOYO study, July 2024, unpublished data).

#### 4.3.3 Individual trajectories

**Figure 6:**
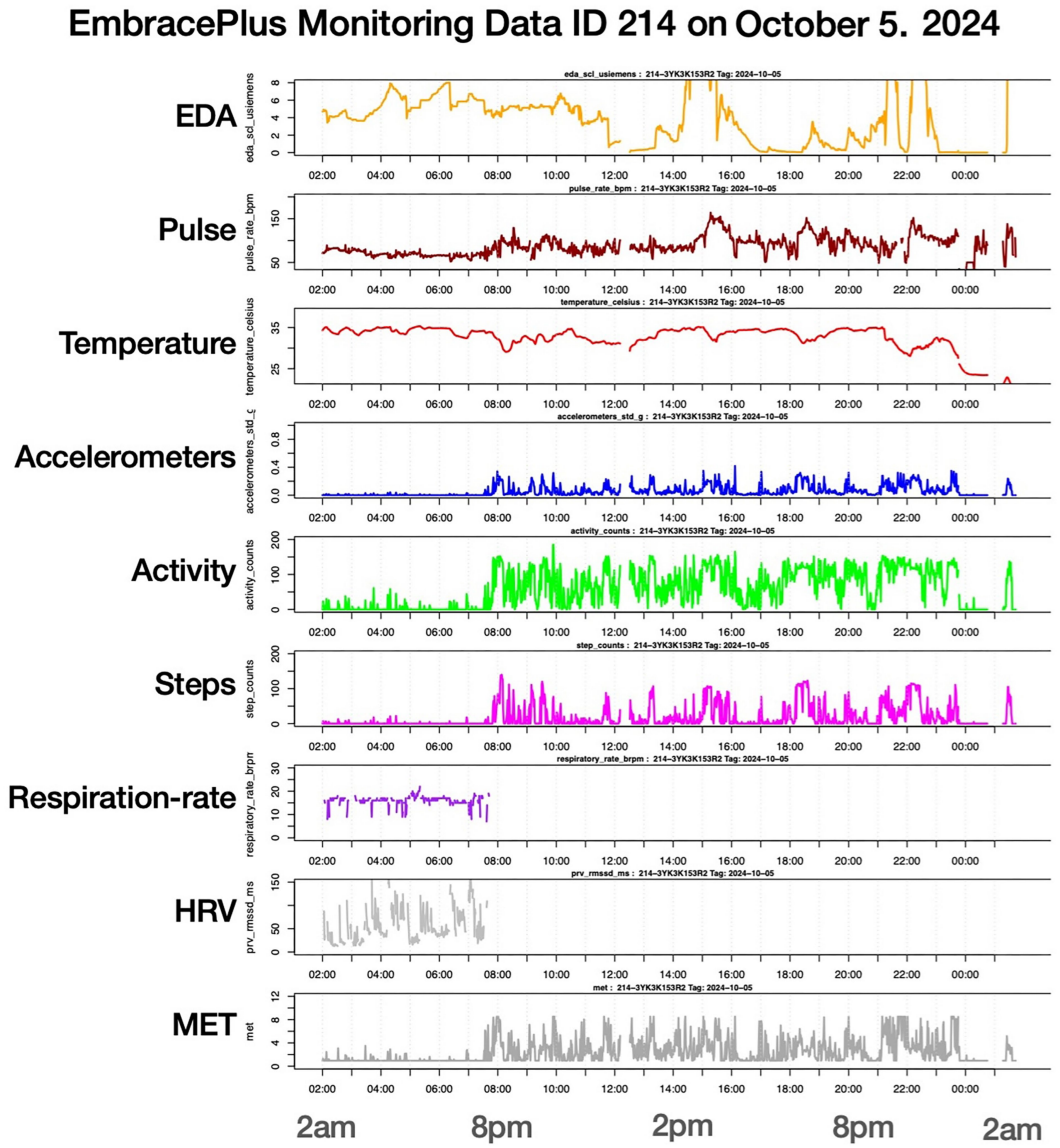
Minute-aggregated smartwatch data for participant ID 214 across the concert day. Stacked panels display electrodermal activity (EDA), pulse rate, wrist temperature, accelerometry, activity counts, step counts, respiration rate, heart-rate variability (HRV), and metabolic equivalents (MET; for abbreviations see Section 3.4). Pronounced elevations in EDA and pulse are visible before the first Elbphilharmonie rehearsal, at concert onset and conclusion, and during post-concert social activity. Respiration rate and HRV (derived from blood-volume pulse) are available primarily during resting periods, whereas movement results in missing values. Note: EDA = electrodermal activity; HRV = heart-rate variability (RMSSD derived from blood-volume pulse); MET = metabolic equivalents; accelerometry reflects tri-axial wrist movement. Data are shown as minute-wise aggregates. Date refers to October 5, 2024 (concert day at the Elbphilharmonie).

Minute-by-minute data for an individual example (ID 214) illustrate day-long co-variation of electrodermal activity (EDA), pulse rate, wrist temperature, and movement (see Figure 6). Because respiration rate and heart-rate variability (HRV) are derived from blood-volume pulse (BVP), these variables are largely unavailable during periods of movement but remain informative during resting phases (e.g., sleep duration and nocturnal recovery). For ID 214, pronounced elevations in EDA and pulse occurred prior to the first rehearsal at the Elbphilharmonie, at concert onset and conclusion, and during post-concert social activities. Interpretation of such elevations requires synchronized activity logs and contextual self-reports to differentiate between physical exertion and emotional or cognitive arousal.

**Figure 7:**
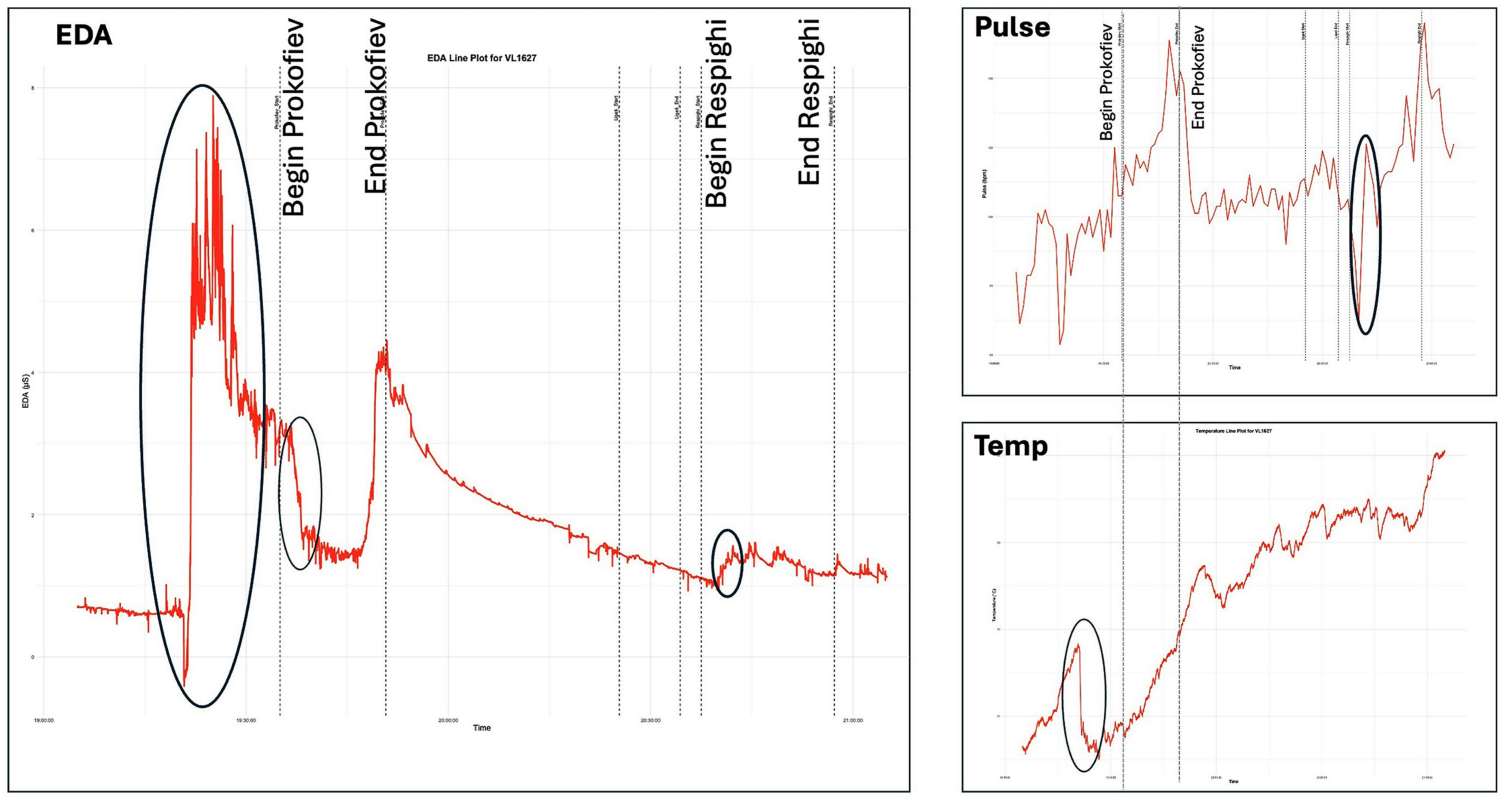
Raw, time-aligned physiological traces of violinist VL1627 during the concert performance of *Sergei Prokofiev: Romeo and Juliet, Suites Nos. 1 & 2 (excerpts)* and *Ottorino Respighi: Pini di Roma* at the Elbphilharmonie (Hamburg, October 5, 2024). Left panel: tonic EDA with dashed lines marking the boundaries between *Prokofiev* and *Respighi*. Right panel: pulse rate (top) and wrist temperature (bottom). An increase in EDA accompanied by a decrease in wrist temperature is observed at stage entry; physiological recovery follows toward the end of *Prokofiev*. A second EDA elevation coincides with the participant-reported “shock moment” at the end of *Prokofiev*. Wrist temperature decreases prior to concert onset, while pulse rate reaches its highest levels before the concert and during the final applause.

A case analysis of violinist VL1627 further illustrates the relationship between physiological markers and subjective experience (see Figure 7). Raw, time-aligned recordings showed a marked increase in tonic EDA accompanied by a concurrent decrease in wrist temperature at stage entry, consistent with strong anticipatory activation. Physiological recovery was observed only toward the end of Prokofiev’s Montagues and Capulets. A subsequent EDA elevation, together with concomitant changes in pulse rate and wrist temperature, coincided with the participant-reported “shock moment” (forgetting to count repetitions) at the conclusion of the Prokofiev movement. Following the lengthy spoken address, EDA rose again at the beginning of the second half of the concert. Wrist temperature showed a pronounced decline prior to concert onset, which the participant attributed to cold hands, while pulse rate reached its highest levels immediately before the concert and during the final applause.

#### 4.3.4 Data scale, quality, and ecological insights

**Figure 8:**
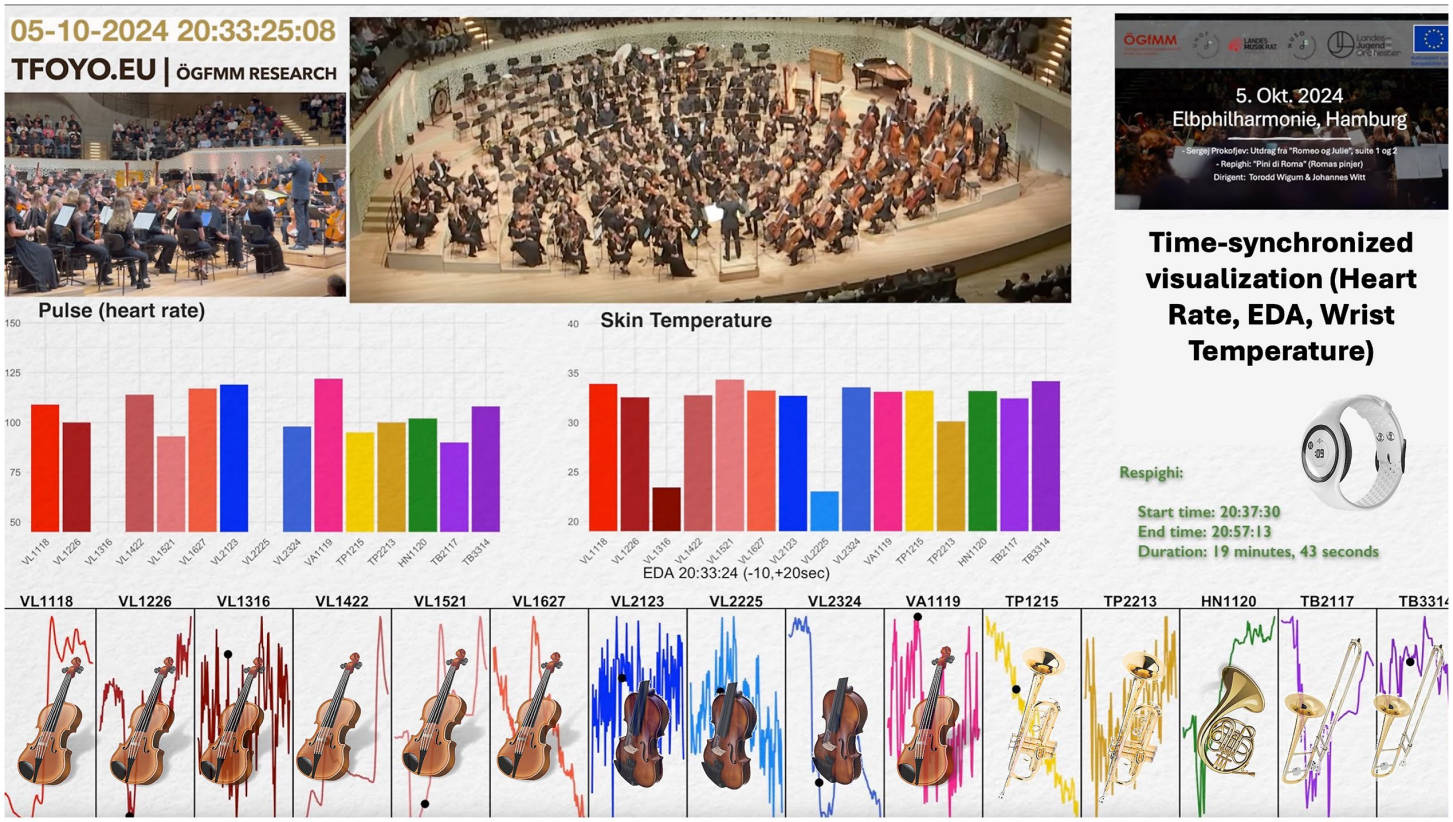
Time-synchronized multimodal visualization (15 musicians; strings and brass) during the concert performance of *Sergei Prokofiev: Romeo and Juliet, Suites Nos. 1 & 2 (excerpts)*, *György Ligeti: Lontano*, and *Ottorino Respighi: Pini di Roma* at the Elbphilharmonie. Composite display linking dual video perspectives and the annotated score with per-player physiological signals (heart rate, EDA, wrist temperature) and rolling 30-s EDA windows. Instrument icons and anonymized player IDs indicate section membership (six first violins, three second violins, one viola, two trumpets, one horn, two trombones). The highlighted timestamp illustrates a *Respighi* segment (20:37:30–20:57:13) and demonstrates second-by-second alignment across modalities.

Each device produced more than 22 million raw samples per day, enabling fine-grained temporal resolution but also increasing susceptibility to motion-related artefacts, particularly when watches were loosely fitted, insufficiently charged, or worn by musicians with highly active arms. Cross-checking physiological signals with tri-axial accelerometry supported artefact identification and the retention of high-quality data segments for visualization and interpretation. Importantly, pronounced physiological elevations were not confined to musically demanding passages: post-concert increases aligned with applause, social interaction, and celebratory activities, underscoring the ecological validity of the recordings. All raw signals were processed offline using custom R scripts; no real-time feedback was provided to participants. In addition, a composite teaching and analysis video synchronizes physiological data from all 15 participants with two camera perspectives and the annotated score, updating a rolling 30-s EDA window at one-second resolution (see video screenshot in Figure 8). The underlying second-by-second animated visualization, displaying the complete dataset across the entire concert, is publicly accessible via the TFOYO.EU project website.

Together, these findings demonstrate the feasibility of time-synchronized wearable stress monitoring in complex live performance environments and provide a reference framework for interpreting physiological responses observed in more controlled exposure settings, such as virtual reality.

### 4.4 Psychological health: virtual reality exposure training (VRET)

#### 4.4.1 Usability and uptake

Participants reported high perceived realism of the virtual performance environment, and many showed an immediate, spontaneous affective response upon first exposure, indicating a strong sense of presence and experiential salience. Initial willingness to try VRET exceeded 90%, indicating a high degree of uptake and acceptance of the format. Within the camp’s tightly scheduled rehearsal context, repeat sessions occurred less frequently, mainly due to limitations imposed by the sequential setup, while participant interest remained high. Notably, despite these time constraints, more than half of the participating musicians chose to engage in the training on multiple occasions and provided predominantly positive feedback regarding its perceived relevance and usefulness.

#### 4.4.2 Physiological stress responses

Across sessions, VR scenarios elicited psychophysiological activation patterns that were comparable in form to those observed during live performance contexts. Episodes of increased arousal were typically characterized by:

- Electrodermal activity (EDA): transient elevations at solo entries or following performance errors.
- Wrist skin temperature: short-term decreases of up to approximately 2 °C, consistent with sympathetic activation.
- Pulse rate: accelerations or brief decelerations temporally aligned with demanding moments.

**Figure 9:**
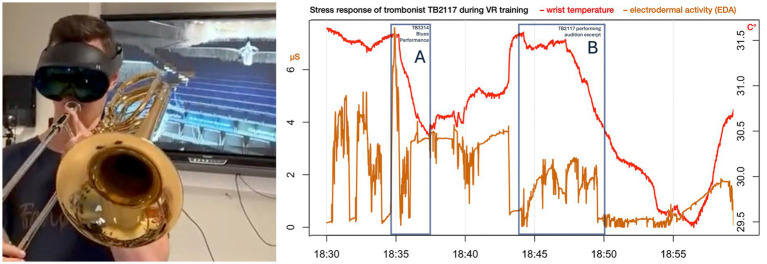
Physiological responses of trombonist TB2117 during virtual-reality performance training. Physiological recordings of an 18-year-old trombonist during a virtual-reality performance session. The graph displays wrist temperature (°C, red) and electrodermal activity (EDA, μS, orange). A: While observing colleague TB3314 performing a big band improvisation (18:35), a decrease in wrist temperature was observed, consistent with anticipatory activation. B: During the participant’s own VR performance at the Elbphilharmonie (18:44), EDA increased and wrist temperature decreased after the initial onset of the Mendelssohn excerpt following a performance error, with a gradual return toward baseline thereafter.

A representative example is shown in Figure 9. While observing colleague TB3314 performing a Big Band improvisation, participant TB2117 exhibited a decrease in wrist temperature, consistent with anticipatory activation. During his own VR performance of a Mendelssohn audition excerpt within the virtual Elbphilharmonie, EDA increased and wrist temperature decreased again after an initial performance error, followed by a gradual return toward baseline. These event-locked patterns mirror physiological responses observed during live concert situations and illustrate the potential of VRET to evoke performance-relevant arousal under controlled conditions.

#### 4.4.3 Data quality and limitations

Smartwatch recordings provided valuable physiological correlates of self-reported arousal, but signals were intermittently affected by motion-related artefacts, particularly due to natural arm movements while holding or playing instruments. Nevertheless, the combined consideration of physiological time series, event-related logs, and contextual self-reports allowed for plausible, time-aligned illustrations of sympathetic activation during virtual reality exposure. These observations should be interpreted descriptively and highlight both the potential and the current technical limitations of wearable monitoring in performance settings.

#### 4.4.4 Subjective benefits

Participants reported that VR training increased familiarity with performance environments, supported anticipation of individually relevant stress triggers, and facilitated reflection on coping strategies for stage fright. The immersive simulations were commonly described as educational and emotionally engaging. While these subjective impressions do not constitute evidence of efficacy, they indicate perceived relevance and acceptability of VRET as a scalable tool for exploratory research and preventive approaches in musician health and resilience training.

### 4.5 Physical health and behavior: practical interventions

The BodyFit and Mental Training workshops were conducted as afternoon group sessions, held after or alongside the intensive rehearsal schedule. Participant motivation was high, with full attendance during the initial sessions. Toward the end of the demanding rehearsal period, attendance declined somewhat, primarily due to time constraints and performance-related stress.

#### 4.5.1 BodyFit workshops

Participants reported immediate improvements in posture awareness, reduction of muscle tension, and enhanced mobility, particularly in high-strain regions such as the shoulders, neck, and lower back. Instructors observed that simple, dynamic mobility and compensation exercises were well received and easily transferable to daily practice. More static posture-correction drills, although effective, were perceived as less engaging. Several participants expressed that they had previously not considered ergonomics in relation to their playing comfort, highlighting the value of introducing these concepts within orchestral settings.

#### 4.5.2 Mental training workshops

Breathing techniques, guided imagery, and short relaxation exercises were readily adopted by participants, who frequently performed them spontaneously before rehearsals and concerts. Coaches observed that these practices enhanced focus, self-confidence, and anxiety regulation. Importantly, offering mental training in a group setting helped normalize psychological preparation, thereby reducing stigma and encouraging open discussion of performance anxiety among peers.

#### 4.5.3 Instructor feedback and subgroup differences

Both instructors emphasized the curiosity and openness of participants toward unfamiliar physical and psychological strategies. The BodyFit coach stressed the importance of early posture and ergonomics training, while the Mental Training coach highlighted the normalization of anxiety-prevention strategies. Cultural and educational subgroup differences were noted: German participants were typically older, often university students, and 25% aspired to professional music careers, whereas Norwegian participants were younger, still in school, and 45.5% indicated professional ambitions. Instructor feedback is reported descriptively and reflects perceived engagement and responses during the sessions; it does not constitute an independent outcome measure.

#### 4.5.4 Overall impact

Both interventions were perceived as highly relevant and practical. They improved short-term physical comfort and psychological readiness, while also laying the foundation for longer-term integration of health-oriented routines into orchestral training.

### 4.6 Survey outcomes

#### 4.6.1 Sample characteristics

A total of 136 musicians participated in the survey component, of whom 134 completed at least one questionnaire. 58 participants provided both pre- and post-camp data. Ages ranged from 13 to 27 years (M = 18.3). The gender distribution was approximately balanced, with 40 male, 52 female, and 10 who did not disclose gender. The instrumental distribution included 36 violins, 8 violas, 14 cellos, 9 double basses, 7 trombones, 3 trumpets, 5 French horns, 2 tubas, 4 oboes, 4 clarinets, 3 flutes, 3 bassoons, 2 harps, 2 percussionists, and 1 piano.

On average, participants had played their main instrument for 10.9 years, had received 9.8 years of individual lessons and 3.4 years of group lessons, and reported about 1.2 h of daily practice (see Table 4).

**Table 4:** Age of participants per intervention group.

Age	BodyFit	Mental training	VR
Valid	48	44	10
Missing	6	9	2
Mean	18.25	18.40	17.8
Std. deviation	3.21	3.03	1.87
Minimum	14	13	15
Maximum	27	27	21

#### 4.6.2 Hearing health and protection behavior

**Figure 10:**
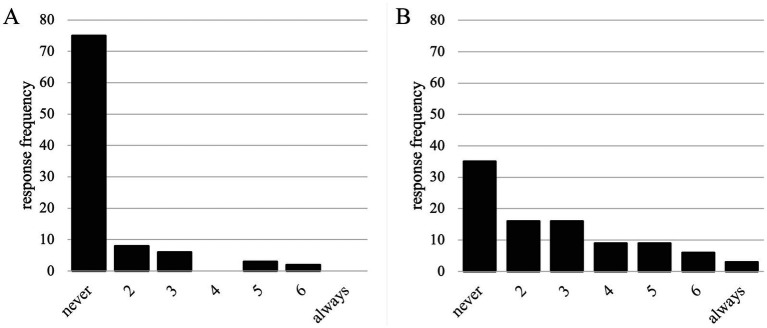
**(A)** How often do participants use hearing protection while practicing alone. **(B)** How often do participants use hearing protection while practicing with an ensemble.

Most participants reported normal hearing, but several disclosed occasional difficulties, such as problems hearing soft sounds or the presence of tinnitus. Hearing protection behavior varied considerably: use was most frequent in solo practice, less common in orchestra rehearsals, and least frequent in concerts (see Figures 10A,B). Only a minority reported consistent use of customized earplugs. Some musicians admitted to using improvised materials such as tissue paper, while others reported no hearing protection at all. These results align with the audiometric screening, where early high-frequency threshold shifts were observed in several brass players. Together, the findings suggest that awareness of auditory risk exists, but consistent protective behavior remains limited.

#### 4.6.3 Coping with stress

93 participants answered the questionnaire regarding their coping mechanisms with stressful situations (SVF-120), for which we instructed them to particularly think of stressful concert situations. Among the positive coping mechanisms, participants scored highest for reaction control and situation control, as well as positive self-instruction, distraction, and need for social support. The highest scoring negative coping mechanisms were rumination, avoidance, and self-accusation. Compared to the SVF’s norm sample, our participants’ mean values were not noticeably above or below average (see Table 5).

**Table 5:** Mean responses over all participants regarding stress coping mechanisms (SVF-120).

*N* = 93	Mean	95% CI mean upper	95% CI mean lower	SD	Minimum	Maximum	T-values acc. to norm sample*
Positive (healthy) stress coping mechanisms							
Overall positive coping	12.731	13.157	12.305	2.069	7.000	17.400	51
Trivializing	11.161	11.836	10.487	3.275	3.000	18.000	47
Downplaying	11.333	12.303	10.363	4.710	3.000	21.000	53
Avoiding liability	10.978	11.753	10.204	3.762	3.000	21.000	52
Distraction	13.452	14.255	12.648	3.902	3.000	21.000	52
Substitute gratification	12.688	13.479	11.897	3.842	3.000	21.000	56
Self-affirmation	11.710	12.488	10.932	3.778	3.000	21.000	51
Relaxation	11.677	12.601	10.754	4.485	3.000	21.000	50
Situation control	14.323	15.152	13.493	4.028	3.000	21.000	45
Reaction control	15.269	15.832	14.706	2.735	8.000	21.000	50
Positive self-instructing	14.720	15.469	13.971	3.637	5.000	21.000	46
Need for social support	13.817	14.823	12.811	4.883	3.000	21.000	52
Negative (unhealthy) stress coping mechanisms							
Overall negative coping	11.403	11.993	10.813	2.865	4.670	18.670	54
Avoidance	13.097	13.902	12.291	3.912	4.000	21.000	52
Flight	10.290	11.202	9.379	4.425	3.000	21.000	54
Social isolation	10.559	11.401	9.717	4.087	3.000	20.000	56
Rumination	14.710	15.567	13.852	4.164	3.000	21.000	50
Resignation	9.957	10.733	9.180	3.770	3.000	21.000	54
Self-pity	10.376	11.138	9.614	3.701	3.000	19.000	52
Self-accusation	12.527	13.325	11.729	3.875	3.000	21.000	53
Aggression	10.075	11.001	9.149	4.497	3.000	21.000	52
Drugs	4.258	4.786	3.730	2.562	3.000	18.000	55

* T-values in relation to the norm sample according to Erdmann and Janke (2008); T-values between 40 and 60 are considered to be average.

#### 4.6.4 Pain and physical health

Participants were asked to rate their current level of pain on a scale from 1–10 regarding 20 different body parts. The pain score is the sum of overall pain reports and thus averaged around 56.37 (*N* = 99, SD = 23.9). Also, participants were asked to report when the pain mostly occurred and how they would cope with it.

Pain was widespread among participants. Across the sample, 8% reported pain “always” while playing, 31% “most of the time,” 57% “rarely,” and only 4% “never.” In another subsample of 108, 25% reported “always” being in pain, 18% “mostly,” 19% “rarely,” and 22% “never.” Pain was most often localized in the neck, shoulders, upper and lower back, and to a lesser extent in the hands and arms; fewer participants reported pain in the legs, feet, jaw, or abdomen. Despite this high prevalence, 32% admitted to practicing even when they knew it would worsen their pain. Almost all (97%) reported no use of medication for pain; among the few who did, typical anti-inflammatory creams or ibuprofen were mentioned.

When asked whom they spoke to about their pain, 25% reported telling their mother, 22% told their music teacher, 19% told their friends, and 18% reported to having told their father, whilst 12% of the participants told nobody about their pain. Of the 88% that spoke about their pain, only 52% felt that their complaints were taken seriously, while 36% felt partially taken seriously. A correlation analysis indicated that higher levels of reported pain were negatively associated with coping strategies involving downplaying or trivializing symptoms, suggesting that musicians experiencing stronger pain were less likely to minimize it.

**Figure 11:**
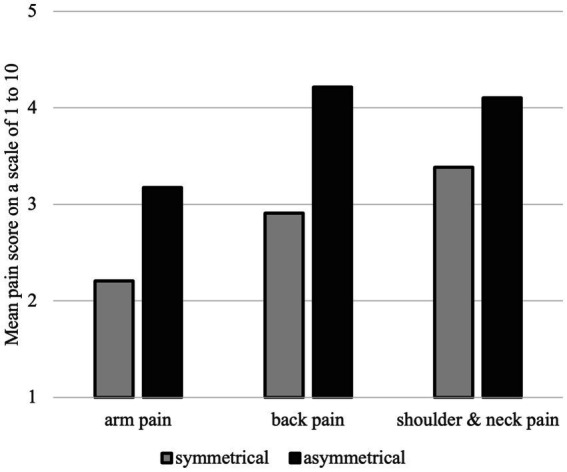
Mean pain score per body region depending on the type of body posture (symmetrical or asymmetrical) required to play the main instrument.

**Figure 12:**
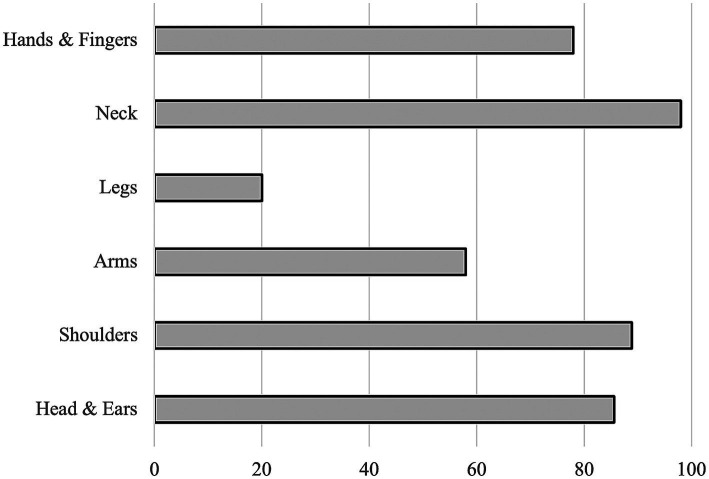
Percentage of participants experiencing pain regularly in the most strained body parts by playing an instrument.

The amount of pain in several body parts was also depending on whether the instrument requires an asymmetrical body posture (see Figure 11). 98% of the participants reported to experience pain regularly. 77% of the young musicians reported to play through the pain (see Figures 11, 12).

Similar findings were reported by Rafal Lawendowski and colleagues in their investigation on study addiction among 132 music students (Lawendowski et al., 2019): Here, too, musculoskeletal pain was identified as a central and widespread health problem among musicians, which is already perceived as burdensome in early adolescence. This study also recognizes problematic practice behavior, in which musicians neglect their physical and social needs, as well as the ignoring of warning signs, with musicians simply continuing to play despite pain.

#### 4.6.5 Performance anxiety and practice behavior

The Music Performance Anxiety Inventory for Adolescents (MPAI-A) indicated that performance anxiety generally increased in the weeks before the concert phase. However, participants in the Mental Training group maintained stable scores across this period, suggesting that the intervention buffered against the typical pre-concert increase.

Self-reported practice-related anxiety was also prevalent: 55% reported anxiety when underprepared, 73% when unable to practice regularly, and 64% described feeling guilty about practicing. Almost half (48%) stated that they had already experienced health problems as a result of instrumental practice. These findings highlight the high psychological load carried by youth orchestra musicians and the extent to which their practice behaviors are intertwined with feelings of anxiety and guilt.

#### 4.6.6 Musical Busyness and coping

A “Musical Busyness” index was calculated based on participants’ reports about the time they spend making music. Concert frequency, rehearsal frequency, and daily practice duration were taken into account. The Musical Busyness score was calculated comparing each participant to their peers by awarding 1 point for being above the sample mean and 1 additional point for each standard deviation that the individual score was above the mean score. For example, the mean practice time was 1.19 h per day with a standard deviation of 0.94, so a score above the 2nd standard deviation of 3.07 h per day would be rewarded with 3 points on the Musical Busyness scale (see Table 6).

**Table 6:** Pearson’s correlations for a priori measured pain level, anxiety, dysfunctional practice behavior, and stress coping mechanisms.

Variable	n/r/p	Pain	Positive stress coping mechanisms	Negative stress coping mechanisms	Dysfunctional practice behavior
Positive stress coping mechanisms	*n*	92	—		
	*r*	0.091	—		
	*p*	0.389	—		
Negative stress coping mechanisms	*n*	92	93	—	
	*r*	0.410***	−0.112	—	
	*p*	< 0.001	0.284	—	
Dysfunctional practice behavior	*n*	92	93	93	—
	*r*	0.407***	0.086	0.065	—
	*p*	< 0.001	0.410	0.536	—
MPA	*n*	92	91	91	91
	*r*	0.374***	−0.237*	0.591***	0.252*
	*p*	< 0.001	0.024	< 0.001	0.016

*p < 0.05, ***p < 0.001.

Participants had an average Musical Busyness score of 3 points and only 25 out of the 94 participants scored a 0, meaning they fell below their peers’ average in all categories. Higher busyness scores correlated negatively with age (Pearson’s *r* = −0.225, *p* = 0.031), indicating that younger musicians were generally more intensively engaged. A very high busyness profile (for example, more than 29 concerts per year, at least three rehearsals per week, and one hour of daily practice) was most common among musicians averaging 16.8 years of age (see Tables 7–9).

**Table 7:** Calculation of Musical Busyness score using mean values and standard deviation among the participants across 5 time-consuming categories.

Practise time [hrs/day]		Concerts per year (ensemble)		Concerts per year (as soloist)		Number of active ensembles		Ensemble rehearsals p. week	
≥ 1.19 h	1P	≥ 12.86	1P	≥ 3.25	1P	≥ 2.66	1P	≥ 1.70	1P
≥ 2.13 h	2P	≥ 22.39	2P	≥ 6.99	2P	≥ 4.43	2P	≥ 2.88	2P
≥ 3.07 h	3P	≥ 31.92	3P	≥ 10.73	3P	≥ 6.19	3P	≥ 4.06	3P

**Table 8:** Musical Busyness score, number of participants with that score and the mean age of this group.

Musical Busyness score	Number of participants with that score	Mean age
0	25	19.13
1	7	17.43
2	10	18.00
3	16	18.67
4	9	18.33
5	10	17.10
6	7	17.29
7 and above	10	16.80

**Table 9:** Mean Musical Busyness score divided by musical instrument.

Instrument	Mean Musical Busyness
Strings	2.34
Woodwinds	3.46
Brass	4.80
Others (*N* = 5)	4.60

**Figure 13:**
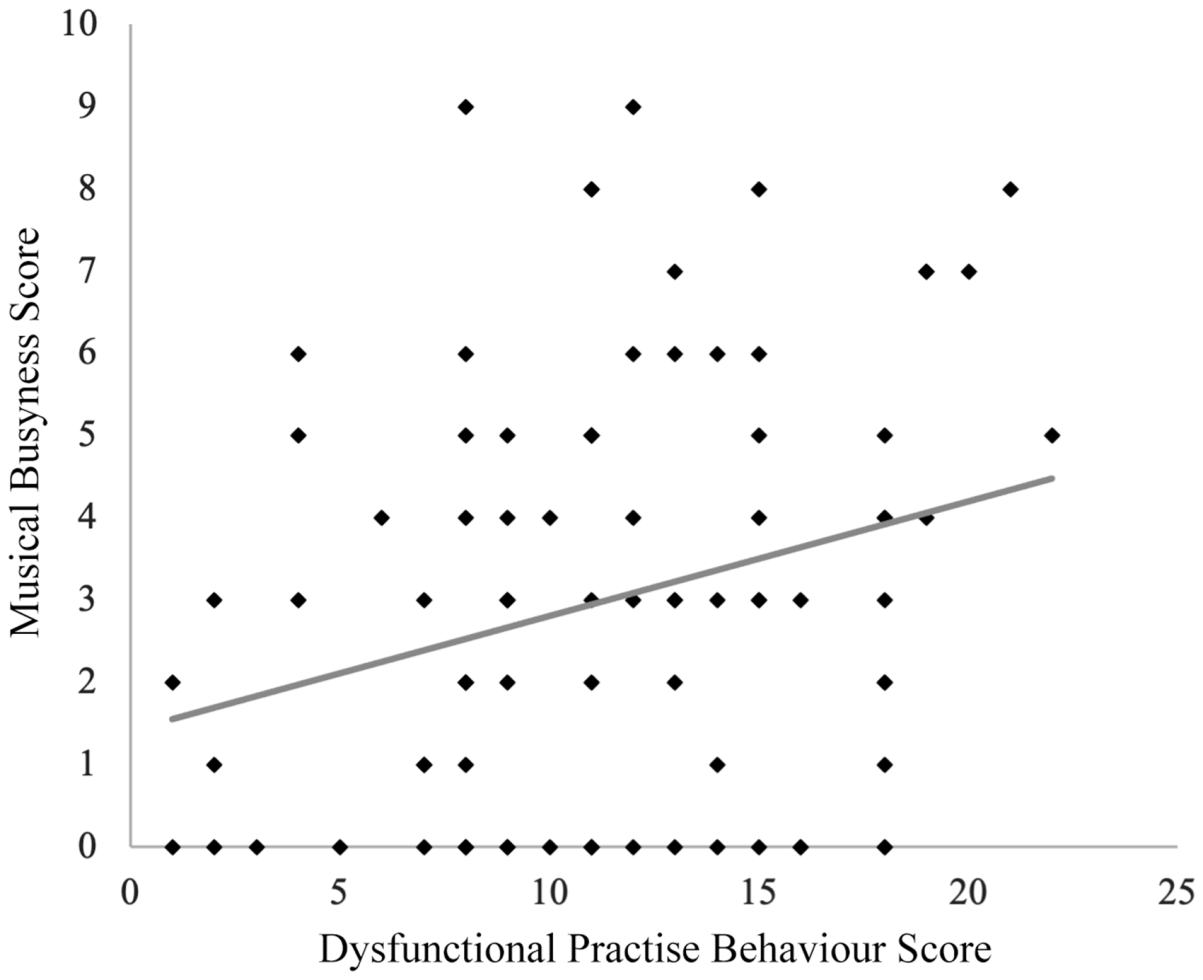
Musical Busyness correlating positively with dysfunctional practice behavior score (Pearson’s *r* = 0.263, *p* = 0.012).

Musical Busyness also varied between instrument groups, and it was linked to psychological functioning. Higher scores correlated positively with positive self-affirmation strategies (Pearson’s *r* = 0.282, *p* = 0.007) and negatively with negative coping strategies (Pearson’s *r* = −0.213, *p* = 0.043). At the same time, higher Busyness was associated with more dysfunctional practice behavior, such as ignoring pain, neglecting social needs, or feeling anxious when not practicing (Pearson’s *r* = 0.263, *p* = 0.012) (see Figure 13). Group comparisons confirmed this pattern: musicians with high busyness engaged significantly less in negative coping (*t* = 2.313, *p* = 0.023, *d* = 0.49) but significantly more in dysfunctional practice (*t* = 2.345, *p* = 0.021, *d* = 0.49).

#### 4.6.7 Professional aspirations

38 young musicians reported to want to become professional musicians. Aspiring professional musicians differed systematically from their peers. They reported significantly higher scores for dysfunctional practice behavior (t_(82)_ = 3.881, *p* < 0.001, *d* = 0.85) and displayed steeper increases in pre-concert anxiety (t_(32)_ = 2.869, *p* = 0.007, *d* = 0.50). This indicates that professional ambition may amplify both the risks of maladaptive practice and vulnerability to performance anxiety.

#### 4.6.8 Intervention-related changes

**Figure 14:**
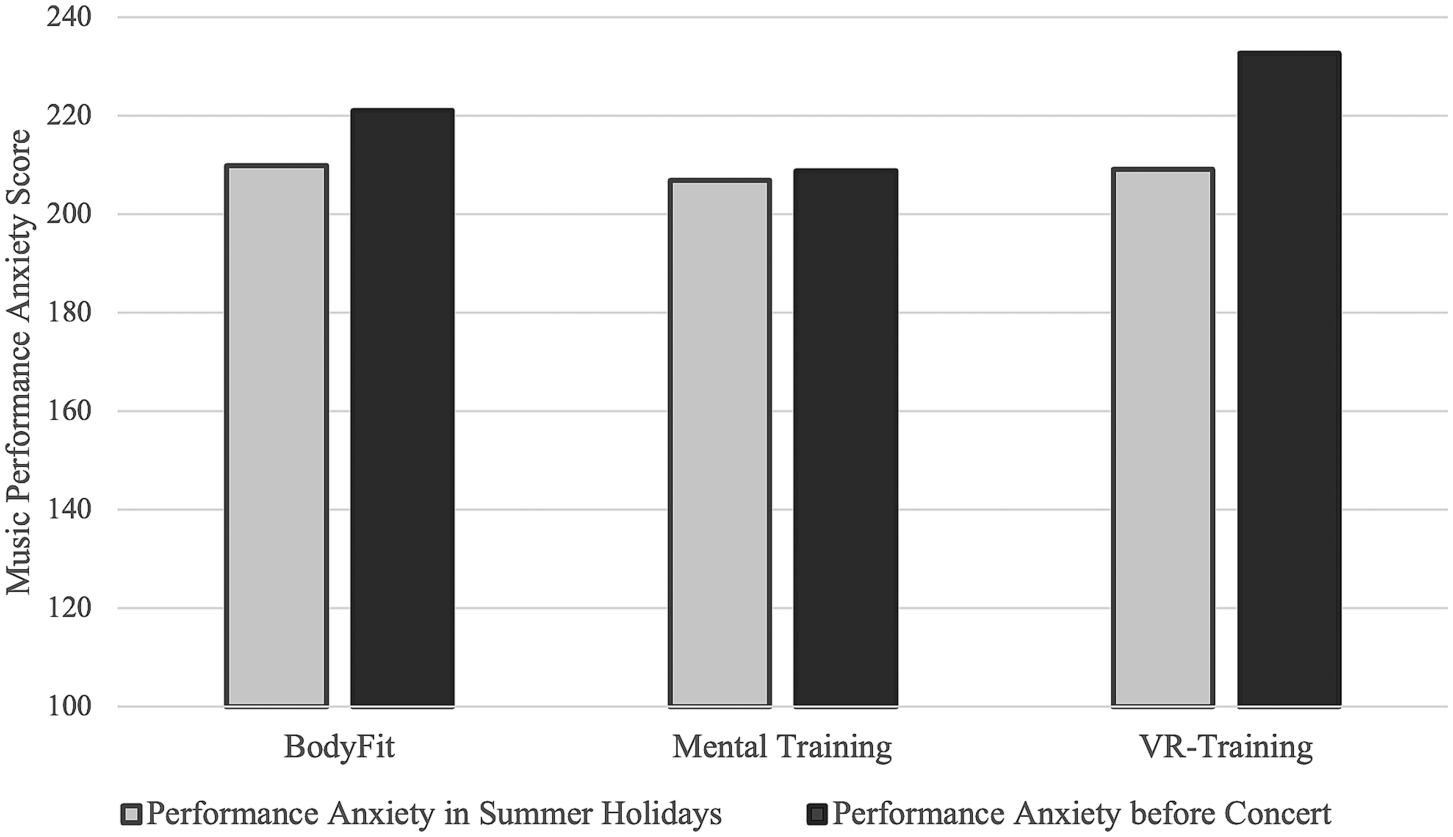
Results of music performance anxiety inventory during the summer holidays and after the intervention, but right before the concert in October grouped by intervention.

**Figure 15:**
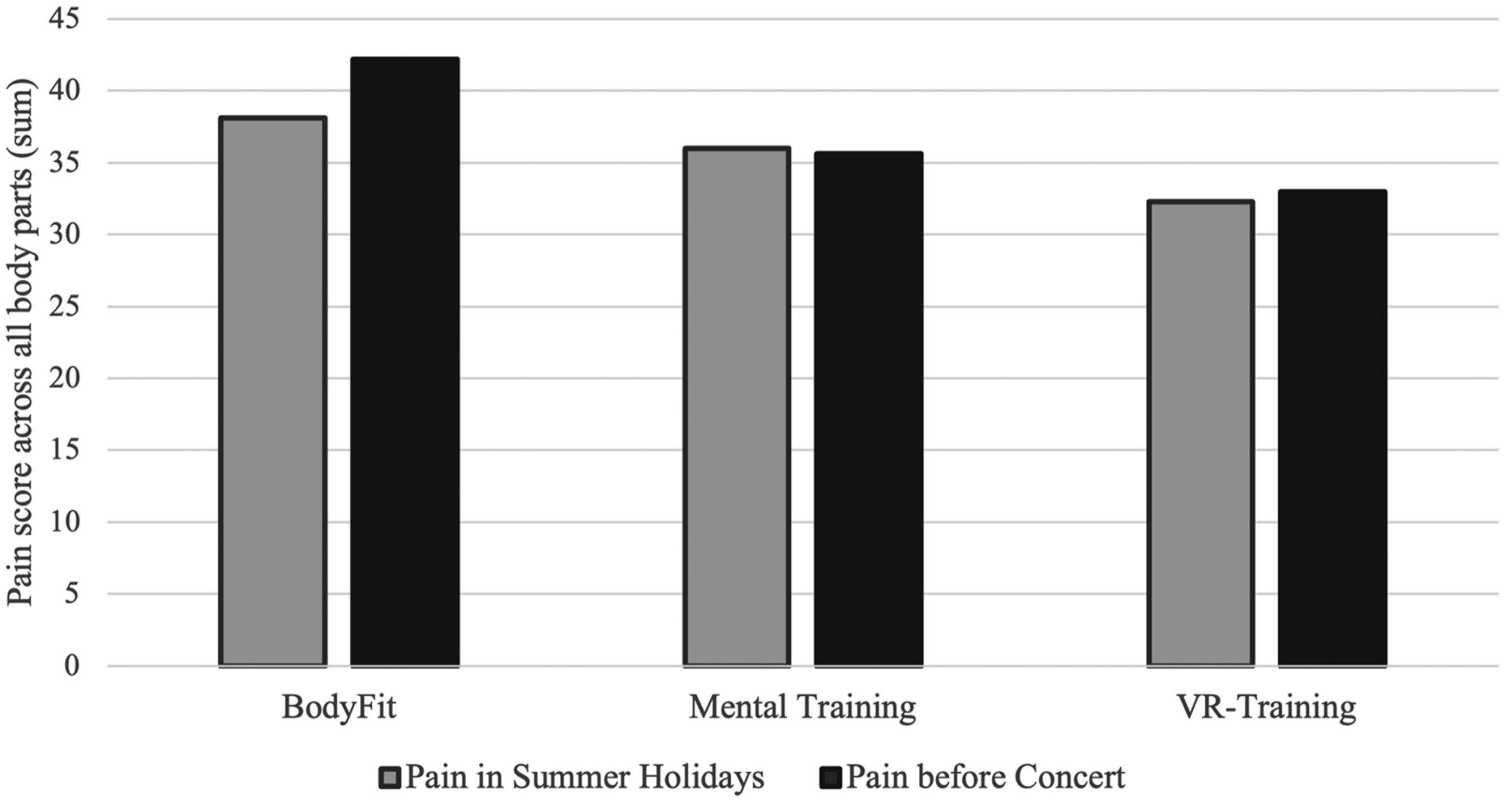
Sum of pain across all body parts during summer holidays and right before the concert in October, grouped by received intervention.

Post-intervention data revealed several patterns. Participants who expressed greater intention to integrate the newly-learned strategies into daily life reported smaller increases in both pain and performance anxiety between camps, though these effects did not reach statistical significance. Among those reporting higher pain levels, improvements in healthy practice behavior were observed (Pearson’s *r* = 0.442, *p* < 0.001). Within the BodyFit group, pain reduction correlated positively with enjoyment of the intervention (Pearson’s *r* = 0.552, *p* = 0.003). Self-reports also indicated greater recognition of the value of physical compensation, more frequent warming up before practice, and increased awareness of warm-up exercises as part of music lessons. However, overall pain scores did not decrease, and in some cases increased slightly, possibly reflecting the dense rehearsal schedule and the timing of the follow-up survey during the beginning of school term and in close proximity to the concert (see Figures 14, 15).

The response rate to the post-intervention interview remained below our expectations, probably also because school and university classes had started for the participants and because the concert was approaching. Total numbers can be found in the Table 10.

**Table 10:** Paired sampled t-tests (pain and anxiety before and after interventions per intervention group).

Group	Mean score before (*N*)	Mean score after (*N*)	*t-*value	df	*p-*value	Cohen’s *d*
Mental training intervention group						
MPA (anxiety)	206.9 (*N* = 38)	208.8 (*N* = 27)	−1.561	21	0.133	−0.333
SD (SE)	45.27 (7.34)	37.0 (7.12)				(SE = 0.135)
∑ overall pain	55.9 (*N* = 40)	55.0 (*N* = 26)	0.423	21	0.677	0.09
SD (SE)	19.94 (3.15)	21.66 (4.25)				(SE = 0.219)
BodyFit intervention group						
MPA (anxiety)	209.9 (*N* = 45)	221.1 (*N* = 31)	−2.050	30	0.049	−0.368
SD (SE)	37.27 (5.56)	37.27 (6.70)				(SE = 0.100)
∑ overall pain	57.9 (*N* = 48)	63.8 (*N* = 26)	−2.498	25	0.019	−0.490
SD (SE)	26.55 (3.83)	27.53 (5.40)				(SE = 0.134)
Virtual reality intervention group						
MPA (anxiety)	209.1 (*N* = 10)	232.7 (*N* = 9)	−1.968	8	0.085	−0.656
SD (SE)	49.25 (15.58)	78.64 (26.21)				0.195
∑ overall pain	51.1 (*N* = 11)	53.25 (*N* = 8)	−3.671	7	0.008	−1.298
SD (SE)	26.42 (7.96)	9.89 (3.5)				0.532

#### 4.6.9 Feedback on the interventions

Quantitative ratings showed that participants evaluated the interventions very positively. On a 7-point scale, mean enjoyment was 5.67, perceived usefulness 5.72, and intention to integrate 5.35. These results underscore the high relevance and feasibility of implementing preventive strategies in real-world rehearsal contexts. The strong willingness to participate, already evident in the initial consent data where fewer than 10% declined participation in any module, was mirrored in consistently high compliance during the camps.

#### 4.6.10 Summary

Overall, the survey outcomes demonstrate that pain, anxiety, and dysfunctional practice behaviors are highly prevalent in youth orchestras, while consistent use of hearing protection remains low. High levels of musical engagement foster adaptive coping strategies but also amplify maladaptive patterns, particularly among those aspiring to be professionals. Interventions such as Mental Training and BodyFit were well received, associated with buffering effects against performance anxiety and improvements in healthy practice behavior among those experiencing pain. These findings highlight both the opportunities and risks of youth orchestra participation: while intense involvement cultivates resilience and self-affirmation, it also increases vulnerability to overuse, auditory risks, and anxiety if preventive strategies are not systematically integrated into training.

## 5 Discussion

### 5.1 Main findings and interpretation

This study suggests that a multimodal health-promotion program can feasibly be implemented in the rehearsal culture of large youth orchestras. Three main findings stand out.

First, regarding hearing health: although most young musicians still exhibited normal audiograms, some brass players already showed early high-frequency threshold shifts. This finding is consistent with previous evidence indicating that the risk of noise-induced hearing loss may begin early in orchestral training and highlights the need for preventive strategies, such as consistent and context-appropriate hearing protection.

Second, concerning physiological stress monitoring: smartwatch-based recordings captured both group-level activation patterns (e.g., anticipatory arousal before rehearsals and concerts) and fine-grained individual stress trajectories linked to solos, performance errors, or unexpected cues. These findings support the ecological sensitivity of wearable monitoring in performance contexts and illustrate its feasibility for linking subjective experiences to objective psychophysiological markers.

Third, regarding practical interventions: structured warm-ups, BodyFit workshops, and mental training sessions were well accepted and widely perceived as beneficial. One pragmatic implementation technique was to carry out the warm-up at the beginning of each rehearsal rather than adding it beforehand, which was associated with improved participation and punctuality. Participants reported increased body awareness and a normalization of psychological skills training, while typical pre-concert increases in anxiety were noted as less pronounced. In line with this, group-level stress-monitoring patterns exhibited transient downshifts in arousal during short on-stage warm-ups or mental focusing exercises before high-stakes run-throughs.

Virtual Reality Exposure Training: VRET elicited physiological stress responses comparable to those observed during live performances, highlighting its potential as a controllable platform for training and exploratory research. Instead of avatar-based or gendered imagery, the training employed photorealistic, 360° scene-captured video of concert halls with ambisonic audio. Participants reported the content as highly realistic and acceptable. The platform was perceived to enhance face validity (i.e., the content looked and felt like the “real thing” to users) and to foster engagement. Adding real-time augmented acoustics may further improve ecological fidelity while preserving experimental control (Bertsch and Frank, 2022; Bertsch and Peschka, 2023).

In classroom deployments, stand-alone VR headsets were rarely plug-and-play in practice, as an initial configuration (guardian/boundary setup, controller pairing, stable Wi-Fi) was usually required. Although all sessions ran successfully, they depended on in-room expertise and small setup buffers. Based on these experiences, we recommend the following implementation measures for educational rollouts: (1) pre-session checks and local content caching, (2) a 10-min setup buffer, (3) a wired or standalone fallback option without casting in low-connectivity scenarios, and (4) basic operator training for staff.

Taken together, these results indicate that targeted health education, preventive interventions, and technology-supported monitoring can be feasibly integrated into youth orchestras and may support resilience and sustainable performance development. A key observation is the modules’ consistently high uptake: over 90% of participants expressed willingness to engage in warm-ups, audiometric testing, and surveys, suggesting that health promotion initiatives are not only feasible but also well received by young musicians.

The survey results further demonstrate that health issues and dysfunctional behaviors are widespread rather than exceptional. This corroborates the initial observations of Britsch (2005), who found that even adolescent orchestra members frequently experienced pain and performance anxiety yet rarely received guidance on prevention or coping strategies. Almost half of the participants reported health problems related to practice, and one-third reported practicing through pain despite knowing it was not advisable.

Musculoskeletal discomfort was almost universal, with over a third of musicians reporting pain “most of the time” or “always.” The pain was most frequently located in the areas of the body that bear the most load when playing an instrument, such as the neck, shoulders, lower back, and hands. Alarmingly, 12% of participants felt that their complaints of pain were not at all taken seriously, revealing a gap between the actual burden of health problems experienced by musicians and the recognition these problems receive in educational contexts. In line with previous studies of competition-level youth musicians (Gembris et al., 2020), most participants reported mild to moderate musculoskeletal discomfort, indicating that PRP may develop prior to tertiary education. These findings reflect the broader spectrum of health and illness in musicians’ medicine, where overuse, psychological stress, and acoustic load interact to cause diseases specific to musicians (Spahn, 2022).

Pain and anxiety stayed the same or increased between the two measurements, but this might be because the first measurement was taken during the summer holidays and the second one right before the concert weeks after the intervention, when school/university had already started for the participants. Also, the response rate was lower than expected, so these results should be treated with caution, as roughly half of the participants responded to both questionnaires in some groups.

### 5.2 Integration across modules

The integration of acoustic, physiological, psychological, and behavioral data revealed important synergies across the different modules. Elevated sound exposure among brass players coincided with early high-frequency hearing threshold shifts and low reported use of earplugs, underscoring the urgent need for targeted hearing-protection education. Conversely, the percussionist(s) who consistently used custom-fit protection showed exceptionally good thresholds, providing a positive counterexample.

Physiological stress markers such as electrodermal activity, pulse, and temperature fluctuations were evident, not only during live concerts, but also in Virtual Reality simulations: validating VR as both an intervention and an experimental paradigm. Arousal peaks observed after concerts were linked to social and celebratory contexts, illustrating that wearable monitoring must be interpreted within the broader ecology of musicians’ lives rather than only in terms of performance demands.

Survey findings on pain, anxiety, and guilt complemented both the audiometric and physiological data. The high prevalence of practicing through pain (32%) aligns with dysfunctional practice patterns and anxiety about insufficient practice (73%), which, in turn, resonate with the stress responses captured before rehearsals and concerts. These converging data illuminate a systemic culture of overpractice and perfectionism that places young musicians at heightened risk.

The linkage between survey-based reports of pain in high-strain body regions, physiological stress responses during difficult passages, and feedback from BodyFit workshops further strengthens the case for integrated interventions. While BodyFit sessions improved awareness of posture and tension, the persistence of pain across the cohort indicates that more sustained or intensive programs may be needed. Moreover, the very low rate of medical treatment uptake highlights an unmet need for structured health education and accessible physiotherapeutic support within youth orchestra programs.

Taken together, the interdependence of the modules shows that the challenges identified (hearing risk, stress reactivity, pain, and maladaptive practice) are mutually reinforcing. This underscores the necessity of a holistic approach to health promotion in youth orchestras, where acoustic, psychological, and physiological dimensions are addressed simultaneously rather than in isolation.

### 5.3 Comparison with existing literature

The present findings are consistent with previous evidence indicating that adolescent musicians already experience musculoskeletal pain, stress, and early-stage hearing problems at rates approaching those reported for professional musicians (Gembris et al., 2018; Schink et al., 2014). Comparable prevalence rates of 60–90% have been reported among professional orchestral musicians worldwide (Blum and Ahlers, 1994; Zetterberg et al., 1998; Abréu-Ramos and Micheo, 2007; Ackermann et al., 2012; Steinmetz et al., 2015; Kenny and Ackermann, 2015; Gasenzer et al., 2017). The underuse of hearing protection observed in this cohort mirrors long-standing challenges documented by Pawlaczyk-Łuszczyńska et al. (2011a, 2011b), pointing to persistent barriers in translating risk awareness into consistent protective behavior.

The observed associations between mental training participation and reduced performance anxiety are in line with earlier reports by Kenny and Osborne (2006), who emphasized the need for age-appropriate psychological support, and by Campbell (2020), who demonstrated the value of implementing mental skills and body-based interventions in youth orchestra training. Early preventive approaches addressing performance anxiety appear particularly relevant, given that even pre-adolescent students exhibit substantial levels of music performance anxiety (Nusseck et al., 2015). Similarly, the VRET-related findings align with prior work (Williamon et al., 2014; Glowinski et al., 2015; Bissonnette et al., 2016), demonstrating the potential of immersive technologies in performance-anxiety research. The present study extends this line of work by illustrating comparable patterns of physiological activation during VR exposure and live performance within a complex, ecologically valid orchestral context.

Although physical health issues among musicians have been widely documented, holistic models of well-being, such as those proposed by the Ecology of Musical Performance (EMP) framework, remain underrepresented in empirical research (Fukumura et al., 2025). This gap is particularly evident for young musicians in pre-professional training contexts.

Finally, the wearable stress-monitoring data provide fine-grained insight into both group-level arousal patterns and individual trajectories, responding to established calls for ecologically valid, field-based psychophysiological measurement in performance research (Williamon et al., 2014) and aligning with recent developments toward more realistic, context-rich performance simulation and monitoring tools (Waddell et al., 2025). Together, these findings situate TFOYO within a growing body of research advocating integrated, multimodal approaches to musicians’ health.

### 5.4 Methodological considerations

Key strengths of this project include its real-world orchestral setting, the integration of multiple data modalities, and the ecological validity afforded by combining rehearsals, concerts, and residential camps. The combination of subjective self-reports with objective physiological and acoustic measures enabled a more nuanced interpretation than would have been possible using a single-method approach. Like recent participatory approaches in health protocol development for young musicians (Shoebridge and Osborne, 2025), the intervention framework was informed by direct input from students and orchestra staff, underscoring the relevance of tailored and co-designed preventive strategies.

Several limitations must be acknowledged. Subsamples within individual modules were relatively small (e.g., VRET participation was limited primarily to brass players), which restricts generalizability. Wearable devices, while offering rich physiological data, were susceptible to motion-related artefacts: EDA signals were affected by arm movements, and wrist skin temperature was sensitive to ambient conditions. Participation was voluntary, and self-selection into specific interventions may have introduced a bias toward participants with greater health awareness or interest. In addition, age, experience level, and professional aspirations appeared to shape outcomes, with musicians pursuing professional careers reporting higher dysfunctional practice scores and more pronounced pre-concert anxiety.

The documentation of consent preferences prior to the study provided valuable insight into participant motivation and supported intervention planning. At the same time, the notability high rates of reported willingness may partly reflect social desirability effects. Future research should therefore complement self-reported measures with longitudinal behavioral indicators to more accurately assess sustained engagement and longer-term impact.

In addition, the intensive rehearsal schedules and limited recovery opportunities inherent to residential orchestra camps posed additional practical constraints. Daily walking distances between venues, combined with extended working hours and prolonged daylight exposure during the Bodø phase, likely affected rest and sleep quality. These contextual factors highlight that, although the multimodal framework proved feasible under field conditions, its implementation must carefully account for musicians’ restricted time resources and cumulative environmental load.

### 5.5 Practical implications and future directions

The present findings inform several practical considerations for promoting health awareness and resilience in youth orchestras:

- Integration into rehearsal culture: Early, structured health interventions such as daily warm-ups can be implemented into orchestral routines with minimal disruption, provided they are aligned with rehearsal schedules and artistic priorities.
- Audiometric screening and hearing education: Noise-risk education and access to appropriate hearing protection appear particularly relevant, especially for high-risk sections (e.g., brass, percussion, high woodwinds). Emphasizing situational use (e.g., during fortissimo passages or close-range exposure) rather than constant wear may support uptake of the intervention; periodic audiological screening can be arranged independently of rehearsal settings.
- Physical and mental skills training: BodyFit and mental training modules may be considered for integration into youth orchestra, conservatory, and music school contexts to support physical awareness and psychological coping skills.
- Virtual reality exposure training (VRET): VR offers a controllable and repeatable exposure setting for stage-fright–related training and research. Future developments could incorporate adaptive features such as real-time augmented audio and additional performance environments.
- Wearable monitoring as pedagogy: Beyond research applications, physiological self-monitoring may serve as a reflective learning tool, helping young musicians recognize and interpret individual stress trajectories in relation to rehearsal and performance demands. For routine use by students and teachers, research-grade devices such as the EmbracePlus are of limited practicality, due to the absence of user-facing data streams and high acquisition costs (approximately USD 3,000). In contrast, consumer wearables (e.g., Apple Watch, Polar, Garmin) offer accessible heart-rate and HRV indicators that are suitable for educational contexts and guided self-reflection. For clinical investigations or research purposes, however, the EmbracePlus’s access to raw data and higher sensor fidelity remain advantageous.

Looking ahead, health awareness could be conceived as a normalized and continuous element of music education, from schools and youth orchestras to conservatory curricula, rather than as an optional supplement.

Overall, the findings suggest that health promotion initiatives in youth orchestras may be particularly promising when coupled with digital innovation and active youth participation. Preventive strategies were perceived as more acceptable when embedded in broader educational and cultural frameworks, indicating that future European collaborations may benefit from pursuing such integrated approaches to support long-term adoption.

### 5.6 Limitations

While the project demonstrated feasibility and promising outcomes, several limitations must be acknowledged:

- Sample size per module: Although 136 musicians participated overall, subgroup analyses (e.g., VRET, BodyFit, Mental Training) were based on smaller samples, limiting statistical power and generalizability.
- Measurement conditions: Physiological monitoring took place in naturalistic but heterogeneous settings. While this enhances ecological validity, it necessarily reduces experimental control.
- Technical artifacts: Wearable data were occasionally affected by motion-related artefacts (EDA) or ambient temperature influences (skin temperature), requiring careful data cleaning and cautious interpretation.
- Self-selection bias: Musicians who opted into specific modules may have been more health-conscious or motivated, potentially inflating levels of engagement and perceived benefit.
- Survey burden: The extensive pre-camp survey battery may have contributed to respondent fatigue, which could have affected response quality or completion rates.
- Generalizability: Findings are specific to intensive residential youth orchestra camps and may not directly extend to other educational settings, age groups, or less immersive training formats.

Despite these limitations, the project provides a coherent multimodal feasibility framework and demonstrates that evidence-informed health promotion activities can be implemented and accepted within youth orchestra contexts. Rather than establishing effectiveness, the findings offer a structured basis for future controlled and longitudinal studies aimed at developing scalable and context-sensitive approaches to musician health.

## 6 Conclusion

### 6.1 Summary

This study shows that combining objective multimodal measurements—acoustic, physiological, and psychological—with a didactic intervention program is feasible and well-accepted in the youth orchestra context. By mapping orchestral soundscapes, screening hearing thresholds, and monitoring stress responses with wearables, as well as integrating structured warm-ups, body conditioning, and mental training, the project established an integrative framework for health promotion. The findings suggest that young musicians reported increased awareness of health-related risks and perceived value in practical strategies to support resilience, coping with pain, and reflection on performance anxiety. Further showing the effectiveness of the interventions at an institutional level, the participating orchestras initiated follow-up measures, including plans to provide filtered hearing protection as standard equipment, where previously only basic foam earplugs had been available.

This multimethod approach enables a differentiated understanding of individual and group needs and may inform targeted preventive strategies early in a musician’s educational trajectory. Beyond the immediate study context, the project generated sustainable educational outputs, including freely accessible instructional videos and informational materials that continue to be used and shared via social media and project channels and have already prompted inquiries from other youth orchestras.

### 6.2 Sustainability

A central strength of the project lies in its commitment to informational sustainability and open knowledge transfer. All developed materials—including daily warm-up routines, the *Get in the Zone* video, photorealistic VR scenarios, interactive online soundscape maps, audiometric tools, and health guidelines—are freely accessible as open resources. This ensures long-term availability for educators, musicians, and institutions beyond the project’s duration.

Informational sustainability was further supported by digital innovation and close collaboration between scientific experts and the leadership structures of youth orchestras. VR modules, training videos and handouts are permanently available online. Furthermore, youth committees and co-creation processes piloted in TFOYO offer a transferable model for integrating health, education and youth empowerment. Collaboration across European professional networks enhanced the project’s impact by enabling the development of context-specific solutions through international partnerships, cultural exchange and adaptation to diverse rehearsal settings. This kind of transnational collaboration makes it possible to share best practice between youth orchestras and professional training environments for young musicians. It provides a scalable framework for implementing evidence-based health promotion in orchestral training in Europe and beyond.

## Data Availability

The datasets presented in this study can be found in online repositories. The names of the repository/repositories and accession number(s) can be found in the article/supplementary material.

## Footnotes

1 The interactive SPL soundscape in the supplementary material can be found at: https://muwiserver.univie.ac.at/tfoyo/.

2 The interactive 360° 3D application of immersive orchestral experience can be found at: https://www.mdw.ac.at/mrm/iasbs/virtuelle-touren/TFOYO/.

3 All audiograms are accessible through an interactive online orchestra layout, enabling instrument-specific exploration of hearing thresholds in the supplementary material at: https://muwiserver.univie.ac.at/tfoyo/audiometrie.
